# QbD based Eudragit coated Meclizine HCl immediate and extended release multiparticulates: formulation, characterization and pharmacokinetic evaluation using HPLC-Fluorescence detection method

**DOI:** 10.1038/s41598-020-71751-y

**Published:** 2020-09-10

**Authors:** Faaiza Qazi, Muhammad Harris Shoaib, Rabia Ismail Yousuf, Fahad Siddiqui, Muhammad Iqbal Nasiri, Kamran Ahmed, Iyad Naeem Muhammad, Farrukh Rafiq Ahmed

**Affiliations:** 1 Department of Pharmaceutics and Bioavailability and Bioequivalence Research Facility, Faculty of Pharmacy and Pharmaceutical Sciences, University of Karachi, Karachi, 75270 Pakistan; 2 Department of Pharmaceutics, Faculty of Pharmacy and Pharmaceutical Sciences, University of Karachi, Karachi, 75270 Pakistan; 3 Department of Pharmaceutics, Faculty of Pharmacy, Hamdard University, Karachi, Pakistan

**Keywords:** Biological techniques, Health care, Medical research, Materials science

## Abstract

This study is based on the QbD development of extended-release (ER) extruded-spheronized pellets of Meclizine HCl and its comparative pharmacokinetic evaluation with immediate-release (IR) pellets. HPLC-fluorescence method was developed and validated for plasma drug analysis. IR drug cores were prepared from lactose, MCC, and PVP using water as granulating fluid. Three-level, three-factor CCRD was applied for modeling and optimization to study the influence of Eudragit (RL100-RS100), TEC, and talc on drug release and sphericity of coated pellets. HPLC-fluorescence method was sensitive with LLOQ 1 ng/ml and linearity between 10 and 200 ng/ml with R^2^ > 0.999. Pharmacokinetic parameters were obtained by non-compartmental analysis and results were statistically compared using logarithmically transformed data, where p > 0.05 was considered as non-significant with a 90% CI limit of 0.8–1.25. The AUC_0–t_ and AUC_0–∞_ of ER pellets were not significantly different with geometric mean ratio 1.0096 and 1.0093, respectively. The C_max_ of IR pellets (98.051 ng/ml) was higher than the ER pellets (84.052 ng/ml) and the T_max_ of ER pellets (5.116 h) was higher than the IR pellets (3.029 h). No significant food effect was observed on key pharmacokinetic parameters of ER pellets. Eudragit RL100 (6%) coated Meclizine HCl pellets have a potential therapeutic effect for an extended time period.

## Introduction

Meclizine HCl, prescribed for both prophylactic and therapeutic management of nausea, dizziness, and vomiting owing to motion sickness, acts as an antagonist on histamine (H1) receptors. It is also used for the treatment of pruritus, Type 1 hypersensitivity reactions as well as vertigo related to disease conditions affecting the vestibular system. Meclizine HCl belongs to the biopharmaceutics classification system (BCS) class II, attributed to low solubility and high permeability^1^. It is currently not available as an extended-release (ER) dosage form. The plasma elimination half-life of Meclizine HCl is 5–6 h^2,3^. For the therapeutic management of vertigo, the approved dose is 25–100 mg per day, administered in two or more divided doses^4^.

In this study Quality by Design (QbD) based Eudragit coated ER Meclizine HCl extruded-spheronized pellet formulations were designed along with immediate-release (IR) core pellets to improve patient compliance whilst reducing the intervals between doses and the risk of dose-dependent adverse and side effects. Poly (meth) acrylate Eudragit polymers type A (RL100) and type B (RS100) are obtained from esters of acrylic and methacrylic acid, bearing functional group trimethylammonioethyl COOCH_2_·CH_2_N + (CH_3_)_3_·Cl, which is responsible for the physicochemical properties. Both polymers are recommended for sustained release formulations. They are water-insoluble and provide pH-independent swelling. RL100 shows higher permeability compared to RS100^5^.

Meclizine HCl contains diphenylmethane chromophore with R_1_ = Cl, R_2_ = N(CH_2_)_2_NH (piperazine moiety), and R_3_ = *m*–C_6_H_4_CH_3_ (Supplementary Fig. S1). The excitation-emission wavelengths reported for Meclizine HCl were 265 (λex) and 291 (λem)^6^. The analytical methods reported for the assessment of Meclizine in human plasma were based on UV-Spectrophotometer^7,8^, UV-HPLC^9–11^, and LCMS/MS/MS^12–14^. No HPLC method along with fluorescence detection technique is previously reported for the determination of Meclizine in human plasma. Imai et al. developed a reverse-phase ion-pair high performance liquid chromatographic method for the quantitation of Meclizine dihydrochloride in serum. In this method, 5 ml of blood was collected per sample and 2 ml serum was used for drug extraction, owing to which it is not suitable for human studies. Secondly, cyclohexane (8 ml) was used as an extraction solvent which is expensive and hazardous for environment^11^. Arayne et al.^9^ reported the HPLC–UV method for the analysis of Meclizine in dosage forms and human serum. The sensitivity of this method was low (LLOQ 25 ng/ml) and selectivity against endogenous plasma peaks was not evaluated. Mismatched matrix (supernatant collected after plasma protein precipitation with acetonitrile, ACN) was used for the preparation of p lasma calibration standards which does not match with the actual bio samples obtained from pharmacokinetic (PK) study^9^. Sher et al.^10^ reported the HPLC–UV method for the simultaneous determination of antihistamine drugs in dosage forms and human serum. Automated solid-phase extraction was used for plasma sample preparation and linearity was observed in the range of 10–2,150 ng/ml. Although, the limit of quantification was below 10 ng/ml for all antihistamines^10^. Liquid chromatography–mass spectrometry (LC–MS) based bioanalytical methods were costly. In line with extant literature, a sensitive and cost-effective alternate for quantification in human plasma by utilizing native luminescence of Meclizine was developed and validated as per FDA and ICH guidelines for precision, selectivity, accuracy, robustness, recovery and stability^15,16^. This HPLC-fluorescence method was used to compare pharmacokinetic (PK) parameters of Meclizine HCl ER pellets with immediate-release pellets. The effect of food on pharmacokinetic parameters of ER pellets was also investigated.

## Materials

Meclizine hydrochloride was a gift from Ali Gauhar Pharmaceuticals Private Limited, Pakistan. Microcrystalline Cellulose, MCC (Avicel PH-101) was obtained from FMC Corporation, USA. Lactose monohydrate, Polyvinylpyrrolidone (PVP, MW ~ 44,000), Talc, Pentanesulphonic, and Heptanesulphonic acid salts were purchased from BDH Chemical Laboratories suppliers, Poole, England. Eudragit RL100 and RS100 were generously provided by Evonik industries, Darmstadt, Germany. All solvents and reagents used were of analytical grade. The assay standards of Meclizine, Cinnarizine, Flunarizine, Pyridoxine, Levofloxacin, Pefloxacin, Ofloxacin, and analytical grade hexane, acetone and isopropyl alcohol (IPA) were purchased from Sigma-Aldrich Chemie GmbH, Germany. Acetonitrile (ACN) was obtained from Tedia Company, Inc., 1000 Tedia Way, Fairfield, USA. Sodium hydroxide was supplied by Merck KGaA, Darmstadt, Germany. Sulphuric acid and hydrochloric acid were provided by VWR Chemicals, BDH Prolabo, USA. Freshly prepared distilled water (DW) was used and all analytical grade reagents and solvents were utilized.

## Methods

### Preparation of IR drug core pellets

Immediate-release core pellets of Meclizine HCl (FC1–FC10) were prepared by extrusion-spheronization technique using variable concentrations of MCC (40–70%), lactose (20–30%) and PVP (0–5%). In a previous study, extended-release matrix pellets of Meclizine HCl were prepared with 60 mg dose^1^. Thus, the amount of Meclizine HCl in IR cores was kept constant at 60 mg. All ingredients were passed through 40 mesh sieve (American Society of Testing and Materials, ASTM) and then weighed. Drug and excipients were dry blended in a planetary mixer for 10 min and then granulated using DW. The wet massing of mixed powder was continued until cohesive and homogenous mass was achieved. To ensure uniform water distribution, sides of the bowl were repeatedly scrapped. Extrusion of wet mass was immediately performed with laboratory-scale mini screw extruder (Caleva Process Solution Ltd, Model. M.S.E, Dorset, UK) fitted with a 1 mm screen at variable speed (50–65 rpm). Collected extrudes were broken down manually into small cylinders. These small cylinders were spheronized (Caleva Process Solution Ltd, Model M.B.S, Dorset, UK) at variable speed (800, 1,000, 1,500, 2,000 rpm) and time (5, 10 and 15 min). The wet pellets were dried in a hot air oven at 40 °C for 12 h^1^.

### Experimental design for ER coating

IR drug cores were coated with Eudragit polymers using Central Composite Rotatable Design (CCRD). The influence of three coating variables polymer, plasticizer, and an anti-tacking agent was determined on four responses drug release, time for 90% drug release (T_90_), aspect ratio, and two-dimensional shape factor (eR). Different grades of Eudragit (RL100 and RS100) were applied to drug cores in a conventional coating pan (ERWEKA, GmbH, Frankfurt, Germany) to evaluate their influence on drug release and sphericity of pellets. To prevent loss of pellets from the pan, 60 mesh sieve was placed on the outer surface of the pan which affected airflow in the pan. The recommended plasticizer, anti-tacking agent, and solvent for Eudragit are Triethyl citrate (TEC), talc, and a mixture of acetone, IPA, and DW, respectively^17^. The relationship between coded and actual values of Eudragit coated pellets and its respective formulations are presented in Table 1. The results of coating variables on responses were analyzed using DESIGN EXPERT version 10. These responses were determined for each formulation and their values were used for modeling and process optimization. Various response surface model (RSM) plots were constructed to understand the influence of variables on responses. The reliability of the model was verified by using ANOVA (Analysis of Variance) with P < 0.05 for each response^18^. The model was selected based on fit summary (lowest SD and press value), ANOVA, and multiple correlation coefficient R^2^ (reasonable agreement between adjusted and predicted values).

**Table 1:** Composition of Eudragit RL100 (FC11–FC30) coating dispersion according to CCRD.

Codes	Space type	Coded values			Actual values (g)					
		RL100	TEC	Talc	RL100	TEC	Talc	IPA	DW	Acetone
FC11	Axial	1.6818	0	0	5.318	0.875	4.375	50.961	4.167	34.304
FC12	Factorial	− 1	− 1	− 1	6.000	0.750	3.750	51.000	4.170	34.330
FC13	Factorial	− 1	1	− 1	6.000	1.000	3.750	50.858	4.158	34.234
FC14	Factorial	− 1	− 1	1	6.000	0.750	5.000	50.288	4.112	33.851
FC15	Factorial	− 1	1	1	6.000	1.000	5.000	50.145	4.100	33.755
FC16	Center	0	0	0	7.000	0.875	4.375	50.003	4.088	33.659
FC17	Center	0	0	0	7.000	0.875	4.375	50.003	4.088	33.659
FC18	Center	0	0	0	7.000	0.875	4.375	50.003	4.088	33.659
FC19	Center	0	0	0	7.000	0.875	4.375	50.003	4.088	33.659
FC20	Center	0	0	0	7.000	0.875	4.375	50.003	4.088	33.659
FC21	Center	0	0	0	7.000	0.875	4.375	50.003	4.088	33.659
FC22	Axial	0	1.6818	0	7.000	0.665	4.375	50.123	4.098	33.739
FC23	Axial	0	1.6818	0	7.000	1.085	4.375	49.883	4.079	33.578
FC24	Axial	0	0	1.6818	7.000	0.875	3.324	50.602	4.137	34.062
FC25	Axial	0	0	1.6818	7.000	0.875	5.426	49.404	4.039	33.256
FC26	Factorial	1	− 1	− 1	8.000	0.750	3.750	49.860	4.077	33.563
FC27	Factorial	1	1	− 1	8.000	1.000	3.750	49.718	4.065	33.467
FC28	Factorial	1	− 1	1	8.000	0.750	5.000	49.148	4.019	33.083
FC29	Factorial	1	1	1	8.000	1.000	5.000	49.006	4.007	32.987
FC30	Axial	1.6818	0	0	8.682	0.875	4.375	49.044	4.010	33.014

Formulations FC31–FC50 comprised of similar composition of Eudragit^®^ RS100 coating dispersions according to CCRD.

#### Eudragit coating on IR drug core pellets

A required amount of Eudragit RL100 or RS100 was homogenized in half of the diluent mixture of IPA, acetone, and DW (57:38:5) for approximately 15 min at 1,500 rpm. TEC and talc were added in a remaining diluent and mixed for 10 min. This dispersion was slowly poured into the Eudragit mixture and stirred for further 15 min. The coating suspension was continuously stirred during the application of coats. The recommended parameters for Eudragit coating were followed: batch size = 50 g, pan speed = 8–10 rpm, nozzle bore = 1 mm, distance nozzle/product = 10–15 cm, atomizing air pressure = 0.8–1.5 bars, spray rate = 1 mL/min, inlet temperature = 40–50 °C, and product temperature = 30–35 °C ± 2 °C. Eudragit coated pellets were dried in a hot air oven at 40 °C for 2 h^17^. The weight gain for Eudragit coating was 5–15%. Dried screened coated pellets were filled manually into 2 size hard gelatin capsule shells. The fill weight was estimated by simply multiplying the tapped bulk density of the pellets with the body volume of the capsule shell. For each capsule size, fill weight was calculated and capsule size 2 was found appropriate for the prepared ER Meclizine HCl coated pellets (tapped bulk density 0.890 g/ml × fill weight 0.37 ml)^19^.

### Drug excipient compatibility

The pure drug, IR cores, and ER coated pellet formulations were subjected to IR spectroscopy using Fourier Transform Infrared (FTIR) spectrophotometer (Thermo Nicolet Avatar, 330) to determine the interaction between active drug and excipients. Spectra were scanned from 4,000 to 500 cm^−1^ wavenumber using OMNIC Spectra Software.

### Characterization of IR core and ER coated pellets

#### Size and flow properties

IR core pellets were sieved using sieve shaker (ERWEKA, Germany) containing a nest of standard sieves for 10 min. Core pellets retained on each sieve were weighed. Size in the range of 2,000–3,000 µm was considered appropriate for ER coating. Flow properties of both immediate and coated pellets were determined by evaluating bulk density, tapped density, compressibility index, Hausner ratio, and angle of repose “α”. These parameters were calculated using the following equations

1 $$Bulk\;density= \frac{M}{{V}_{o}}$$

2 $$Tapped\;density= \frac{M}{{V}_{f}}$$

3 $$Compressibility\;index= \left(\frac{{V}_{o}- {V}_{f}}{{V}_{o}}\right) \times 100$$

4 $$Hausner\;ratio= \frac{{V}_{o}}{{V}_{f}}$$

5 $${tan}_{\left(\propto \right)}= \frac{height}{0.5\;base}$$

where M is the mass of pellets, V_o_ and V_f_ are the bulk and tapped volumes of pellets respectively. Compressibility index 11–15 and Hausner ratio 1.12–1.18 show good flow properties, whereas, compressibility index ≤ 10 and Hausner ratio 1.00–1.11 exhibit excellent flow properties. The angle of repose shows the good flow from 31 to 35 and excellent flow from 25 to 30^20^. Bulk and tapped volumes were measured in triplicate.

#### Moisture content and friability

The water content of pellets, immediately after spheronization and drying was determined. Samples were placed in Petri dishes and heated to 40 °C in a hot air oven until the moisture content (MC) became constant. For friability, 10 g of pellets were placed in a friabilator wheel (ERWEKA GmbH D-63150, Heusenstamm, Germany) and subjected to falling shocks at 25 rpm for 4 min. The 250 µm mesh was used to remove fines and the friability was calculated by remaining above fraction

6 $$Friability \left(\%\right)= \frac{\left(initial\;weight-final\;weight\right)}{initial\;weight} \times 100$$

Friability of less than 1% is considered acceptable. Each batch was analysed in triplicate^21^.

#### Shape and area

The shape and area of each pellet batch (n ≥ 50) were evaluated using stereomicroscope (Am Scope Digital, LED-1444A, USA). These digitalized images were further analysed by image analysis software (NIH Image J 1.47v, USA). Area, perimeter, Feret diameter of the digitalized images were measured and shape factors were calculated as follows:

7 $$\mathrm{Circularity}\;(\mathrm{C})=4\mathrm{\pi A}/{\mathrm{P}}^{2}$$

8 $$\mathrm{Projection\;sphericity}\;(\mathrm{PS})=4\mathrm{A}/\uppi {d}_{L}^{2}$$

9 $$\mathrm{Aspect\;ratio}\;(\mathrm{AR})={\mathrm{d}}_{\mathrm{max}}/{\mathrm{d}}_{\mathrm{min}}$$

10 $${d}_{ce}= \sqrt{\frac{4A}{\pi }}$$

11 $${Shape\;factor\;(e}_{R})=\frac{2\pi }{P}\frac{{r}_{e}}{f}-\sqrt{1-{\left(\frac{b}{l}\right)}^{2}}$$

12 $$Correction\;factor\;(f)=1.008-0.231\left(1-\frac{b}{l}\right)$$

13 $$Elongation\;ratio= \frac{width\;of\;pellets}{height\;of\;pellet}$$

where *A* is the area, *P* is the perimeter, *d*_*max*_ and *d*_min_ are the longest and shortest feret diameters respectively, *d*_*ce*_ is circle equivalent diameter, e_R_ is two-dimensional shape factor, *r*_*e*_ is the mean radius, *f* is a correction factor, *b* and *l* are breadth and length of the pellet, respectively. *b* ˂ *l* elliptical pellets (aspect ratio 1.2–1.5), *b* = *l* round pellets. The limiting value for e_R_ and aspect ratio is 0.6 and 1.1, respectively^22,23^.

#### Surface morphology

External and internal morphology of pellets were visualized using a scanning electron microscope, SEM (JSM-6380A, Jeol, Japan) at 10 kV. IR cores and ER coated pellets were sliced using a razor blade by holding pellets with the help of forceps to evaluate the thickness of the coated polymer layer^24^. Both intact pellets and their cross-sections were placed on aluminum stubs and sputter-coated with gold up to 250 Å using an auto coater (JFC-1500, Jeol, Japan). Photomicrographs were obtained at a magnification ranging from 50 to 1,500 times. Size (dimensions) of pellets and coat thickness was determined.

#### Elemental characterization

Energy dispersive spectrometer (EDS) attached with SEM was used to characterize elements present on the surface and across a section of pellets at 20 kV accelerating voltage. It is a non-destructive technique in which pellets and their cross-sections were bombarded with an electron beam, resulting in the emission of X-rays that are specific to each element. These emitted X-rays were detected by X-ray spectrometer^1^.

### Drug content analysis

Twenty capsules from each batch were randomly selected. The capsule contents were pulverized using mortar and pestle. Sample solution of 10 µg/ml was prepared by utilizing mean weight equivalent quantity in the mobile phase containing 1.5 g of sodium 1-heptane sulfonate in a mixture of DW (300 ml) and acetonitrile (700 ml) at pH 4 (adjusted with 0.1 N sulfuric acid). The samples were sonicated, filtered, and then injected. Signals were detected at 232 nm. The assay was carried out using the C18 column (25 cm × 4.6 mm) with 5 μm packing on HPLC (LC-10AT VP, No.C20973806986 LP, SHIMADZU Corporation, Kyoto, Japan). The mean and standard deviation of three readings from each batch was used^20^.

### In vitro drug release

Meclizine HCl release was determined using USP Apparatus I six-station (ERWEKA DT600, Heusenstamm, Germany) in 0.01 N HCl (900 ml) maintained at 37 ± 0.5 °C at 100 rpm. Dissolution samples (10 ml) were drawn at every 1-h interval over 12 h for ER pellets, whereas, for IR core pellets at 5, 10, 15, 20, 25, 30, 45, 60, 90, and 120 min. Sink condition was maintained by immediately replenished volumes with fresh medium. Collected samples were filtered and 8.4 ml of the filtered aliquot was taken in a 50 ml volumetric flask and made-up volume with drug release medium. The final concentration was 10 µg/ml $(60\;{\text{mg/}}900\;{\text{ml}} \times 8.4\;{\text{ml/}}50\;{\text{ml}})$. In addition to 0.01 N HCl, the release of Meclizine HCl was also determined at pH 1.2, phosphate buffer pH 4.5 and 6.8 for IR cores and optimized ER pellets to determine the influence of pH.

### Drug release kinetics

#### Model-dependent methods

Various kinetic models including Zero-order (Eq. 14), First-order (Eq. 15), Higuchi square root (Eq. 16), Hixson–Crowell cube root (Eq. 17), Baker–Lonsdale (Eq. 18), Jander’s equation (Eq. 19) and Korsmeyer–Peppas (Eq. 20) were applied to in vitro release data of Meclizine HCl to determine its release kinetics using MS Excel (DD Solver)

14 $${Q}_{t}={K}_{0}t$$

15 $$log\;{Q}_{t}=log\;{Q}_{0}+{K}_{1}\frac{t}{2.303}$$

16 $$Q_{t} = K_{H} t^{{{\raise0.7ex\hbox{$1$} \!\mathord{\left/ {\vphantom {1 2}}\right.\kern-\nulldelimiterspace} \!\lower0.7ex\hbox{$2$}}}}$$

17 $$\sqrt[3]{{Q}_{0}}-\sqrt[3]{{Q}_{t}}={K}_{HC}t$$

18 $${\raise0.7ex\hbox{$3$} \!\mathord{\left/ {\vphantom {3 2}}\right.\kern-\nulldelimiterspace} \!\lower0.7ex\hbox{$2$}}\left[ {1 - \left( {1 - {\raise0.7ex\hbox{${M_{t} }$} \!\mathord{\left/ {\vphantom {{M_{t} } {M_{\infty } }}}\right.\kern-\nulldelimiterspace} \!\lower0.7ex\hbox{${M_{\infty } }$}}} \right)^{{{\raise0.7ex\hbox{$2$} \!\mathord{\left/ {\vphantom {2 3}}\right.\kern-\nulldelimiterspace} \!\lower0.7ex\hbox{$3$}}}} } \right] - {\raise0.7ex\hbox{${M_{t} }$} \!\mathord{\left/ {\vphantom {{M_{t} } {M_{\infty } }}}\right.\kern-\nulldelimiterspace} \!\lower0.7ex\hbox{${M_{\infty } }$}} = K_{BL} t$$

19 $$1 - \left( {1 - {\raise0.7ex\hbox{${M_{t} }$} \!\mathord{\left/ {\vphantom {{M_{t} } {M_{\infty } }}}\right.\kern-\nulldelimiterspace} \!\lower0.7ex\hbox{${M_{\infty } }$}}} \right)^{{{\raise0.7ex\hbox{$1$} \!\mathord{\left/ {\vphantom {1 3}}\right.\kern-\nulldelimiterspace} \!\lower0.7ex\hbox{$3$}}}} = K_{J} t^{{{\raise0.7ex\hbox{$1$} \!\mathord{\left/ {\vphantom {1 2}}\right.\kern-\nulldelimiterspace} \!\lower0.7ex\hbox{$2$}}}}$$

20 $${\raise0.7ex\hbox{${M_{t} }$} \!\mathord{\left/ {\vphantom {{M_{t} } {M_{\infty } }}}\right.\kern-\nulldelimiterspace} \!\lower0.7ex\hbox{${M_{\infty } }$}} = Kt^{n}$$

where, *Q*_*t*_ is the amount of drug released in time *t*, *Q*_*0*_ is the initial amount of drug in the sample, *M*_*t*_ is the amount of drug released in time *t*, *M*_∞_ is the amount at infinite time, *M*_*t*_/*M*_∞_ is the fractional solute released,* t* is the time in h, *t*_1/2_ is the square root of time and *K*_0_*, K*_1_*, K*_*H*_*, K*_*HC*_*, K*_*BL*_*, K*_*J*_ and *K*_*KP*_ are the release rate constants for Zero-order, First-order, Higuchi, Hixson–Crowell cube root, Baker–Lonsdale, Jander’s equation, and Korsmeyer–Peppas models, respectively. *n* is an exponent that characterizes the different release mechanisms and calculated through a slope of the straight line.

Drug release was further characterized by determining the mean dissolution time (MDT) and dissolution efficiency (DE) using the following equations:

21 $$MDT=\frac{\sum_{j=1}^{n}{\widehat{t}}_{j}\Delta {M}_{j}}{\sum_{j=1}^{n}\Delta {M}_{j}}$$

22 $$D.E = \frac{{\int_{0}^{t} {y \times dt} }}{{y_{100} \times t}} \times 100$$

where *j* is the sample number, *n* is the number of dissolution sample times, ${\widehat{t}}_{j}$ is the time at the midpoint between *t*_*j*_ and *t*_*j−*1_, ∆M_j_ is the additional amount of drug dissolved between *t*_*j*_ and *t*_*j−*1_ and *y* is the drug dissolved (percentage) at time *t*^1^.

#### Model-independent method (dissolution profile comparison)

The similarity in dissolution profiles was compared by determining the similarity factor (*f*_2_):

23 $${f}_{2}=50\times log\left\{{\left[1+\left(\frac{1}{n}\right){\sum }_{t-1}^{n}{\left({R}_{t}-{T}_{t}\right)}^{2}\right]}^{-0.5}\times 100\right\}$$

where *R*_*t*_ and *T*_*t*_ are the amounts of drug released from the reference and test formulations at each time point, respectively, *n* is the number of dissolution samples. Release profiles are considered different if *f*_2_ < 50^1^.

### Stability studies

Optimized ER formulation means a formulation that achieves desired properties of the target profile i.e. spherical pellets releasing (≥ 90%) drug over an extended period of time (12 h). Optimized ER pellet formulations were studied at 40 ± 2 °C/75% ± 5% RH (relative humidity) for accelerated stability for 6 months, in line with guidelines of International Conference on Harmonisation (ICH)^25^. Encapsulated pellets were placed in amber glass bottles and stored in a humidity chamber (Nuaire, USA). Samples were drawn every 3 months and their drug content, physical appearance, and release characteristics in different media were determined. Software Minitab (version 17.1.0) was used to calculate the shelf-life. The mean of the three samples of each formulation was used to calculate the shelf-life of the coated pellet formulations.

### Method development for quantification of Meclizine HCl

A new high-performance liquid chromatography method with fluorescence detection was developed and validated for the determination of Meclizine in human plasma according to FDA and ICH guidelines^15,16^.

#### Mobile phase preparation

The mobile phase was prepared by dissolving 1.5 g of 1-heptane sulfonate in 300 ml of DW and then 700 ml acetonitrile was added. The pH of the mobile phase was adjusted to 3 with 0.1 N sulphuric acid. The mobile phase was filtered through a 0.45 µm filter and sonicated for 30 min. For complete elution of drug without any interference with plasma peaks, the different proportions of buffer and acetonitrile were tried and pH (3 and 4) of the mobile phase was altered to find out the most appropriate combination of the mobile phase. Two salts 1-pentane sulfonate and 1-heptane sulfonate were also explored for their suitability.

#### Stock and working solutions in the mobile phase and plasma

Standard stock solution 1 mg/ml of the drug was prepared in the mobile phase. From this standard stock solution, a 10 µg/ml solution was prepared in the mobile phase which was then diluted to 1 µg/ml in plasma. Subsequent dilutions of 200, 160, 120, 80, 40, 20, and 10 ng/ml were prepared in plasma from a working solution of 1 µg/ml. Quality control (QC) samples of 180, 100, and 30 ng/ml were also prepared in the same way from a different stock solution.

Plasma samples were pre-treated by four different procedures including liquid extraction with a mixture of Hexane: IPA (9:1), liquid extraction in two steps, double extraction, and protein precipitation. In the case of liquid extraction, each plasma sample (500 µl) was added with 50 µl of 1 N sodium hydroxide (NaOH) and 1 ml of extraction solvent in the ratio of 1:2 (Plasma: Extraction solvent). Samples were vortexed for 1 min and then centrifuged for 10 min at 6,149 × *g*. The upper layer was separated (0.8 ml) and subjected to drying in a vacuum concentrator (Eppendorf concentrator plus, model 5305, Hamburg, Germany). The residues were reconstituted with 500 µl of the mobile phase and transferred to glass vials for injection. For liquid extraction in two steps, extraction solvent (1 ml) was added in two steps to the plasma and the two aliquots were combined and dried under vacuum. The residues were reconstituted with 500 µl of the mobile phase, vortexed, and transferred into HPLC glass vials for injection. In the case of double extraction, the upper organic layer was collected after vortexing and centrifugation of the plasma samples and then transferred in a separate Eppendorf tube containing 500 µl of 0.1 N hydrochloric acid. Samples were again vortexed for 1 min, centrifuged for 10 min, and the lower layer was separated for injection. Simple protein precipitation was carried out with acetonitrile in different ratios to plasma (1:1, 0.9:1 and 0.8:1). The samples were vortexed and then centrifuged. The upper layer (0.4 ml) was collected for injection.

Different drugs that exhibit natural fluorescence like Cinnarizine, Flunarizine, Pyridoxine, Levofloxacin, Pefloxacin, and Ofloxacin were examined as an internal standard (IS) with Meclizine. Both Cinnarizine and Flunarizine were analysed at their respective excitation-emission wavelengths Exλ = 245 nm Emλ = 310 nm and Exλ = 230 Emλ = 292 nm and at the excitation-emission wavelengths of Meclizine Exλ = 265 Emλ = 291 nm. Pyridoxine solution (1 µg/ml) in the mobile phase was analysed at the fluorescence wavelength of Meclizine. Levofloxacin solution (50 µg/ml) in acetonitrile was analysed at Exλ = 295 nm Emλ = 500 nm. Pefloxacin and Ofloxacin (50 µg/ml) in a diluent (ACN: Distilled water, 1:1) were analysed at Exλ = 295 Emλ = 500 nm and Exλ = 285 nm Emλ = 460 nm respectively.

#### Chromatographic conditions

The chromatographic separation of Meclizine was achieved by HPLC (LC-20A, SHIMADZU, Kyoto, Japan) consisting of auto-sampler (SIL-20A), isocratic pump (LC-20AD, Prominence), column oven (CTO-20A), fluorescence detector (RF-20A Prominence) fitted with guard column (Waters, XTerra, RP 18, 5 µm) and column (Mediterranea Sea 18, 5 µm, 25 × 0.46). LabSolutions software version 5.65 (SHIMADZU Corporation) was used for chromatographic data acquisition and analysis. The mobile phase flow rate was set at 1 ml/min. The column temperature was maintained at 40 °C while the autosampler temperature was set at 25 °C. The excitation-emission wavelengths Exλ = 265 Emλ = 291 nm were used for Meclizine (Channel 1) and Exλ = 285 Emλ = 460 nm for Ofloxacin (Channel 2). 50 µl sample was injected onto the column. The observed retention time for Meclizine was 6.2 ± 0.2 min. The RSD (relative standard deviation) for replicate injections was NMT (not more than) 2.0%.

### Method validation

The developed method was validated in terms of selectivity, sensitivity, linearity, accuracy, precision, robustness, and stability as per FDA and ICH guidelines^15,16^. The accuracy of a newly developed method was determined by five replicate analysis of Meclizine plasma samples in a concentration ranging from 10 to 200 ng/ml. For intraday and interday precision, five different concentrations (10, 30, 100, 180, and 200 ng/ml) were evaluated for three consecutive days using the proposed method. The robustness of the developed HPLC-Fluorescence method was determined by deliberately changing some chromatographic variables such as flow rate, pH, mobile phase composition, and excitation-emission wavelengths, and their influence on chromatographic responses like assay and retention time were investigated. One factor was changed at one time without randomization to estimate the effect. Each factor selected was changed at three levels (− 1, 0, + 1) with respect to optimized parameters. The robustness of the method was done at the concentration levels 10 ng/ml and 50 µg/ml for Meclizine and internal standard Ofloxacin respectively. Calibration curves were constructed and the coefficient of correlation (R^2^) was calculated using linear regression by the least square method. SD, RSD, and % CV were determined for all concentrations. Limit of quantification (LOQ) and limit of detection (LOD) were determined by analysing plasma samples of five different concentrations i.e. 0.05, 1, 2, 5, and 10 ng/ml. The response seven times of the noise was considered satisfactory for LLOQ and signal to noise ratio of 3 was considered acceptable for LLOD.

Stability studies in different environmental and expected sample exposure conditions like freeze–thaw, long-term, and stock solutions were conducted. Stock solution stability and long term stability in plasma were investigated for 6 weeks to cover the expected duration of study from the first day of sample collection till the last day of analysis. For post-preparative stability, Meclizine and Ofloxacin samples in autosampler over the anticipated run time was also determined by comparing concentrations with the calibration standards. For analytical recovery, Meclizine concentrations spiked in plasma 10 ng/ml and higher 200 ng/ml were compared with the same concentrations spiked in the mobile phase.

### Pharmacokinetic studies of Meclizine HCl pellets

Encapsulated core pellets formulation containing 60 mg of Meclizine HCl was selected as IR formulation whereas, encapsulated FC12 pellets formulation (coated with 6% Eudragit RL100, 0.75% TEC and 3.75% talc) was chosen as ER pellet formulation for comparative pharmacokinetic study in healthy human volunteers. The study protocols (IBCPH-02) were approved by the Institutional Bioethics Committee (IBC) of the University of Karachi, Karachi, Pakistan. The study was conducted at a single center in Karachi, Pakistan according to the ethical principles provided in the Declaration of Helsinki under the supervision of principal investigator and physician.

#### Inclusion and exclusion criteria

Thirty (30) healthy human volunteers (age 20–25 years, weight 70–74 kg, and BMI 19–25 kg/m^2^) were selected and enrolled for the study. All selected volunteers were physically examined (weight, height, B.P., temperature, pulse rate) along with routine laboratory (blood biochemistry, hematology, urine analysis and pregnancy test for female volunteers) and diagnostic tests (electrocardiogram). Subjects not meeting standard clinical laboratory ranges were excluded from the study. None of the selected volunteers consumed alcohol, tobacco, and nicotine. None of them had any known drug allergies. Subjects were also excluded if they cannot avoid medicines (OTCs, prescribed, vitamin, and mineral supplements) caffeinated beverages or grapefruit during the treatment period. None of the participants received any investigational product or participated in any other pharmacokinetic study within the last 3 months. Volunteers were excluded if they were hospitalized within 8 weeks prior to the study initiation or have donated blood within 30 days before the study.

#### Declaration of consent

All volunteers were explained about the objectives, methods, potential hazards associated with the participation and information regarding the right to withdraw at any time without jeopardy. The subjects were informed verbally and in writing about the consequences and possible outcomes of the study. Both English and Urdu (native language) consent declaration forms were prepared and signed by each volunteer. Participants were instructed to report the occurrence of any adverse effects during the study, washout period, and at the end of the study.

#### Study design

The comparative pharmacokinetic was a single-center, single-dose, open-label, randomized, two-treatment, two-sequence, four-period, cross-over study, comprised of two parts with a separate group of volunteers for each part. In each part of the study, volunteers were assigned to the reference and test groups in a ratio of 1:1. Both treatments were given to each participant in either sequence one or sequence two, separated by a 7 days washout period. Vital signs like B.P., body temperature, heart rate of participants were monitored at the baseline, during, and at the end of each period to assess tolerability.

#### Part 1: Single dose IR (60 mg) vs. ER (60 mg) pellets

This study compared Meclizine HCl IR core pellets with the ER pellets in fasted volunteers. Treatment A was 60 mg Meclizine HCl IR pellets under fasted condition. Treatment B was 60 mg Meclizine HCl ER pellets under fasted condition.

#### Part 2: Single dose ER (60 mg) pellets food effect

This study compared Meclizine HCl ER pellets in fasted vs. fed volunteers. Treatment A was 60 mg Meclizine HCl ER pellets under fasted condition. Treatment B was 60 mg Meclizine HCl ER pellets under fed condition.

In the fasted condition, Meclizine HCl IR or ER encapsulated pellet formulations were administered to volunteers after an overnight fast of ≥ 10 h in the morning with plain water (240 ml). Subjects remained in the fasted state after 4 h of the dose. In the fed condition, Meclizine HCl ER encapsulated pellet formulation was administered to volunteers after overnight fasting of ≥ 10 h in the morning and 30 min after the start of a standardized high-calorie (800–1,000 cal) and high-fat breakfast meal. The breakfast meal consisted of two butter-fried eggs, two beef strips, two toast slices with butter, potatoes (four ounces), milk (eight ounces). Caffeine and xanthine drinks were prohibited.

#### Blood sampling

Venous blood samples (5 ml) were drawn from the antecubital vein of each volunteer by direct venepuncture, before administration at 0 h and after dosing at 0.5, 1, 2, 3, 4, 5, 7, 9, 12, 18, 24, 36 and 48 h. Samples were collected into vacutainer tubes containing anticoagulant sodium citrate. Plasma was separated within 30 min of collection by centrifugation for 10 min at 6,149 × *g*. These plasma samples were transferred into propylene tubes and kept frozen at − 40 °C till analysed.

### Bioanalysis, pharmacokinetics and statistical analysis

Meclizine HCl concentration in plasma was analysed and measured using a newly developed and validated HPLC method with fluorescence detection. The pharmacokinetic parameters of part 1 and part 2 studies were calculated using PK software KINETICA version 5.1 (Thermoelectron, corp., Waltham bbb, USA) for non-compartmental analysis. Statistical analysis was performed using a two-way analysis of variance (ANOVA) and a two-one-sided t-test. The analysis was performed on log-transformed data as per FDA guidelines^26^. The factors sequence, period, and treatment in the model were fixed effects and subject within the sequence was a random effect. Geometric mean ratios (GMRs) and corresponding 90% CIs were determined for ER pellets/IR pellets (Part 1 study) and ER pellets under fed/fasted conditions (Part 2 study). Bioequivalence was claimed if the resulting 90% CIs were within the prespecified boundaries (0.800–1.250 or 80–125%).

## Results and discussion

### IR drug core pellets

#### Influence of formulation variables

The IR core pellets of Meclizine HCl (FC1–FC10) were prepared by extrusion-spheronization. MCC is a well-reported and accepted extrusion-spheronization aid for the synthesis of pellets as it retains water like a sponge and prevents separation of water from the solid blends during processing, thus, desired rheological properties, cohesiveness, and plasticity for spherical pellets are achieved^27^. In this research, among different formulation variables granulating fluid has an important role to play in the preparation steps of pellets. Water as a granulating fluid bound powder blend during wet massing and facilitated the extrusion process by its lubricating and plasticizing characteristics^28^. MCC formed fragile Meclizine HCl pellets (FC2, 70% MCC) only at higher water level (45–50%) because the growth of agglomerates during pelletization occurred at certain moisture levels by coalescence. MCC has the capability to absorb water, hence, an increased amount of MCC required more water for the growth of agglomerates^29^. The yield was less because extrudes were not appropriately spheronized. Less amount of granulating fluid was required for pelletization when lactose was added in the formulation to increase the strength of the pellets^30^ but these pellets were irregularly shaped (FC4, 40% MCC, 30% lactose). Thus, the quantity of water required for pelletization was dependent on characteristics and concentrations of formulation ingredients^28^. This lactose based irregularity in the shape of the pellets was also reported by other researchers^31^. Therefore, the amount of lactose was reduced from 30 to 20%, and MCC was increased to 50% in FC5 (50% MCC and 20% lactose). Dumbbell shape pellets were formed only at a higher level of granulating fluid (31–35%), even, in the presence of 50% MCC (FC6). In all these formulations extrudes were formed very slowly irrespective of the extrusion speed.

**Figure 1:**
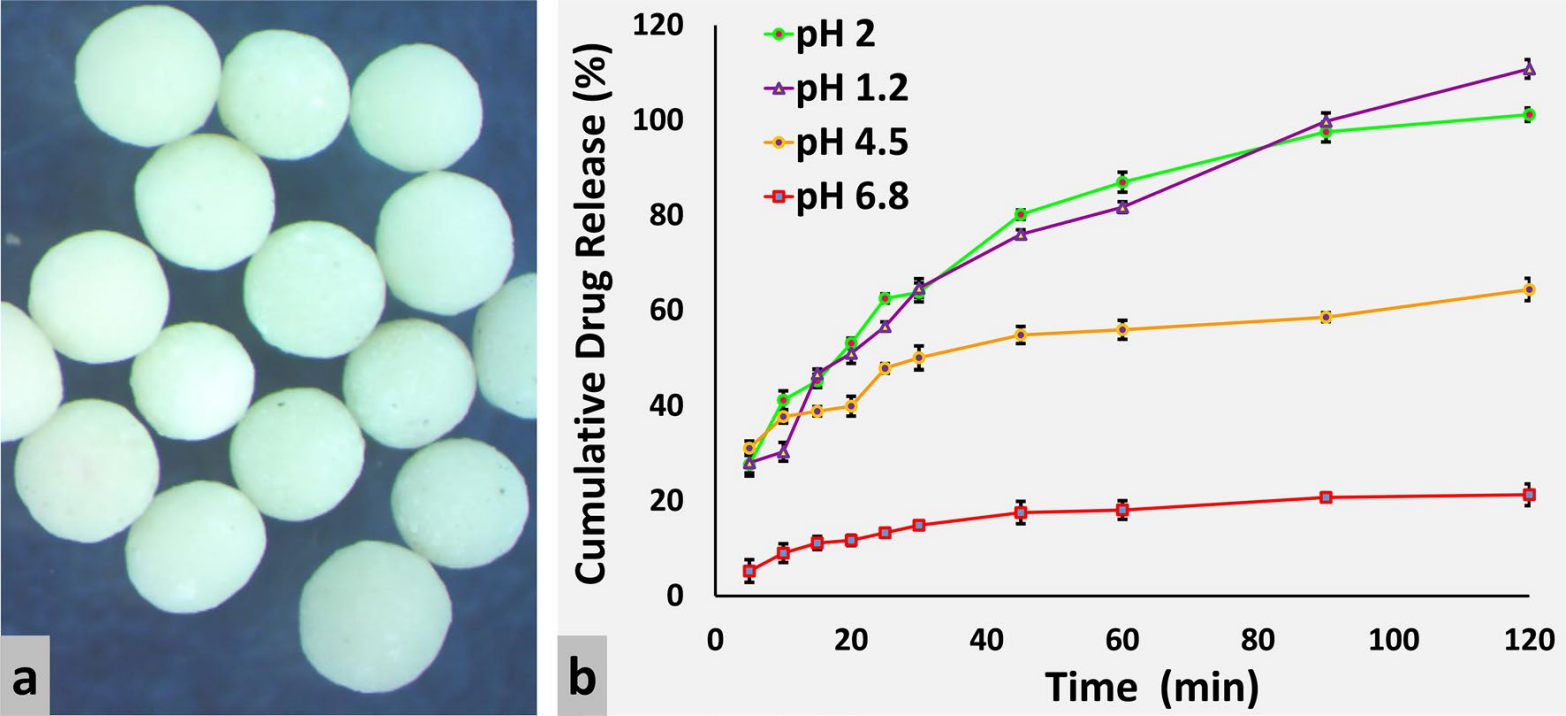
(**a**) Stereo microscopic images and (**b**) in vitro release profiles of Meclizine HCl IR core pellets at different pH.

These irregularly shaped pellets of Meclizine HCl were made spherical by the addition of PVP (2%) in FC7 (48% MCC, 20% lactose, and 2% PVP). PVP, due to its low adhesive strength provides necessary plasticity and lubrication to the wetted mass, hence required less amount of granulating fluid, promoted extrusion rate, and produced pellets with a spherical shape and enhanced yield (89%). Extrudes were also formed rapidly in the presence of PVP and remained independent of the amount of granulating fluid (FC8). When the amount of PVP was increased up to 5% in FC9 (45% MCC, 20% lactose, and 5% PVP), spherical pellets were produced with maximum yield (92%) by utilizing 20–25% granulating fluid, illustrated in Fig. 1a. Spherical and hard pellets (FC10) were also produced with the same composition at a higher level of granulating fluid (26–30%). PVP is a synthetic binder polymer which bound ingredients of pellets to enhance the strength of the agglomerates, extrudability and to maintain the integrity of pellets^32^. MCC-PVP also produced spherical pellets with different types of Eudragit^28^.

#### Influence of process variables

Beside different formulation ingredients, production variables also influenced the process of pelletization. Different extrusion speeds (50, 55, 60, and 65 rpm) were employed to determine their effect on the preparation of pellets. The formation of extrudes remained independent of the extruder speed. However, the residence time in spheronizer and its speed had a pronounced effect on the sphericity of Meclizine HCl IR core pellets. At low spheronizer speed (600 rpm) for 10 min, the force of shearing was minute, forming compact bread by breaking short cylinders. Higher speed (1,000–1,500 rpm) resulted in the formation of spherical pellets with a large number of fines, even, at a residence time of 3 min because centrifugal force was generated which causes cracking of pellets^33^. Therefore, extrudes were spheronized at low speed (800 rpm) for an initial 2 min to convert them into uniform, small granules by the circular motion of the friction plate. Later, speed was increased (1,500 rpm) for the remaining 3 min to convert small granules into spherical pellets by the collision of granules between the plate and walls of the spheronizer. Thus pellet sphericity is a function of spheronization speed and time^34^.

#### Sphericity and release from Meclizine HCl IR core pellets

Stereo images of IR core pellets FC9 are shown in Fig. 1a. The combination of MCC and PVP formed spherical and smooth surface pellets of Meclizine HCl having aspect ratio and shape factor values ranged from 1.010 to 1.040 and 0.980–0.991, respectively. The shape factor value was increased with a higher concentration of PVP. The release of Meclizine HCl from core pellets was investigated to determine release kinetics and mechanism. The adapted target profile for the IR formulation of Meclizine HCl was not less than (NLT) 75% after 45 min in 0.01 N HCl^20^. Since drug core pellets (FC9) released 80% Meclizine HCl after 45 min (Fig. 1b), therefore, met the criteria and selected for extended-release coating.

### Eudragit coated pellets

#### *Drug release from* Eudragit *coated pellets*

**Figure 2:**
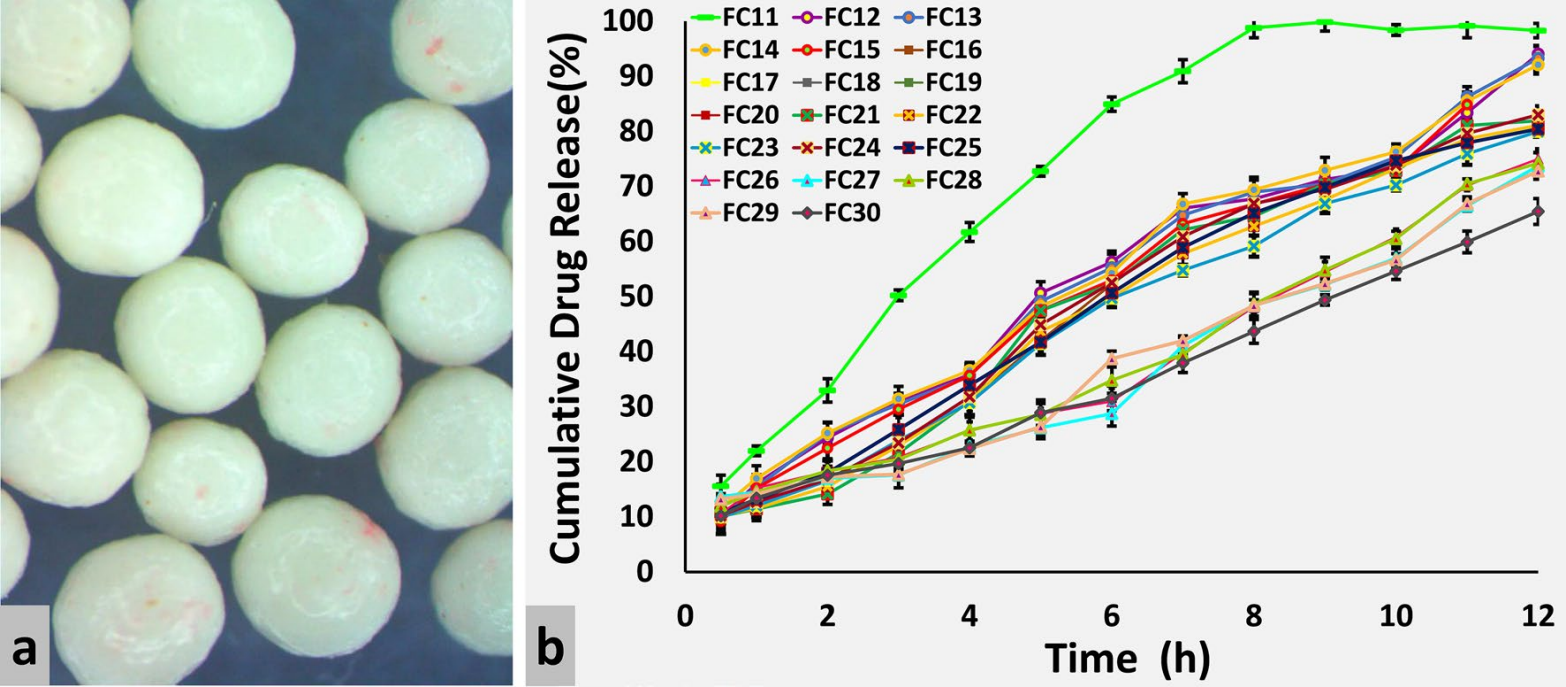
(**a**) Stereo microscopic images and (**b**) in vitro release profiles of ER Eudragit RL100 coated pellet formulations FC11–FC30.

**Figure 3:**
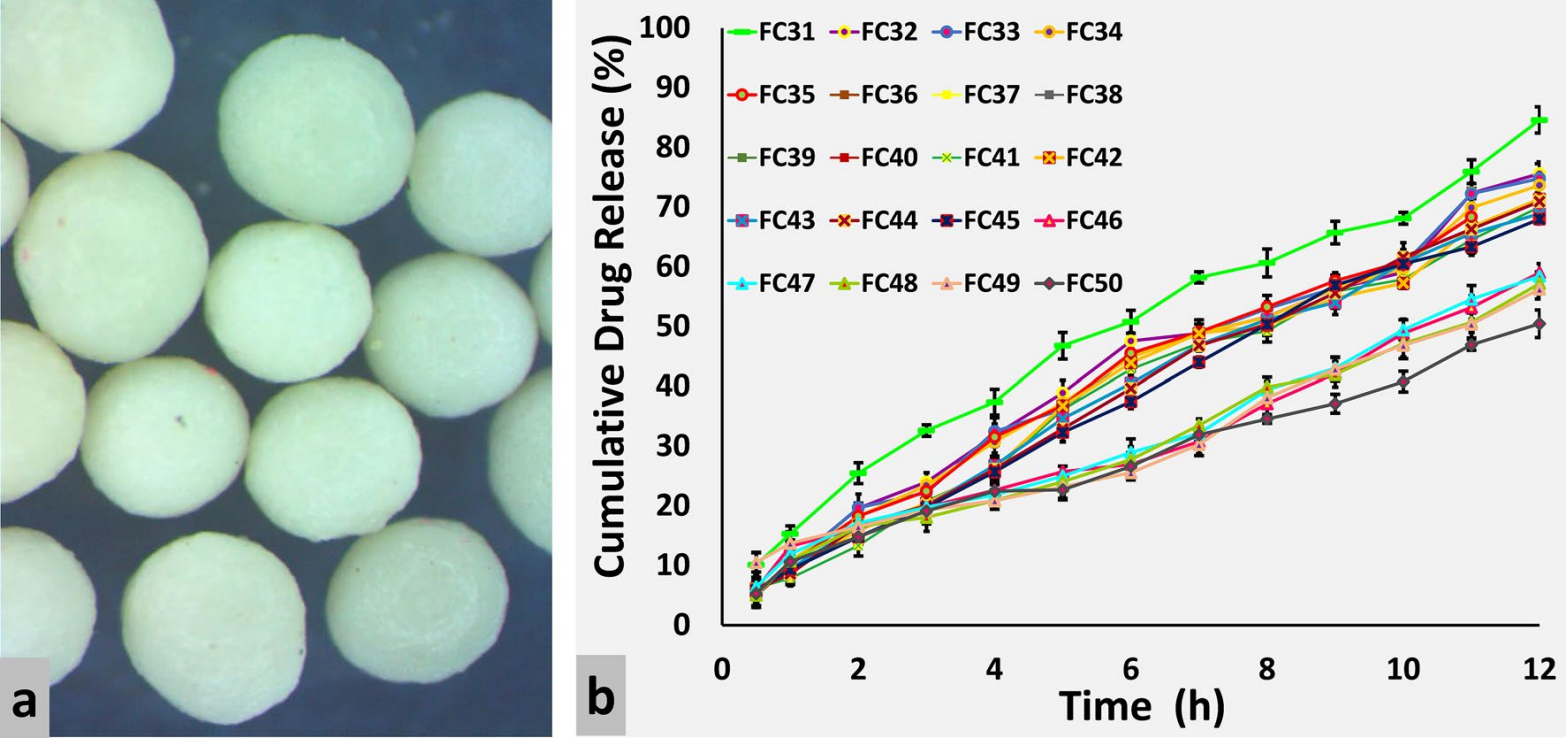
(**a**) Stereo microscopic images and (**b**) in vitro release profiles of ER Eudragit RS100 coated pellet formulations FC31–FC50.

**Figure 4:**
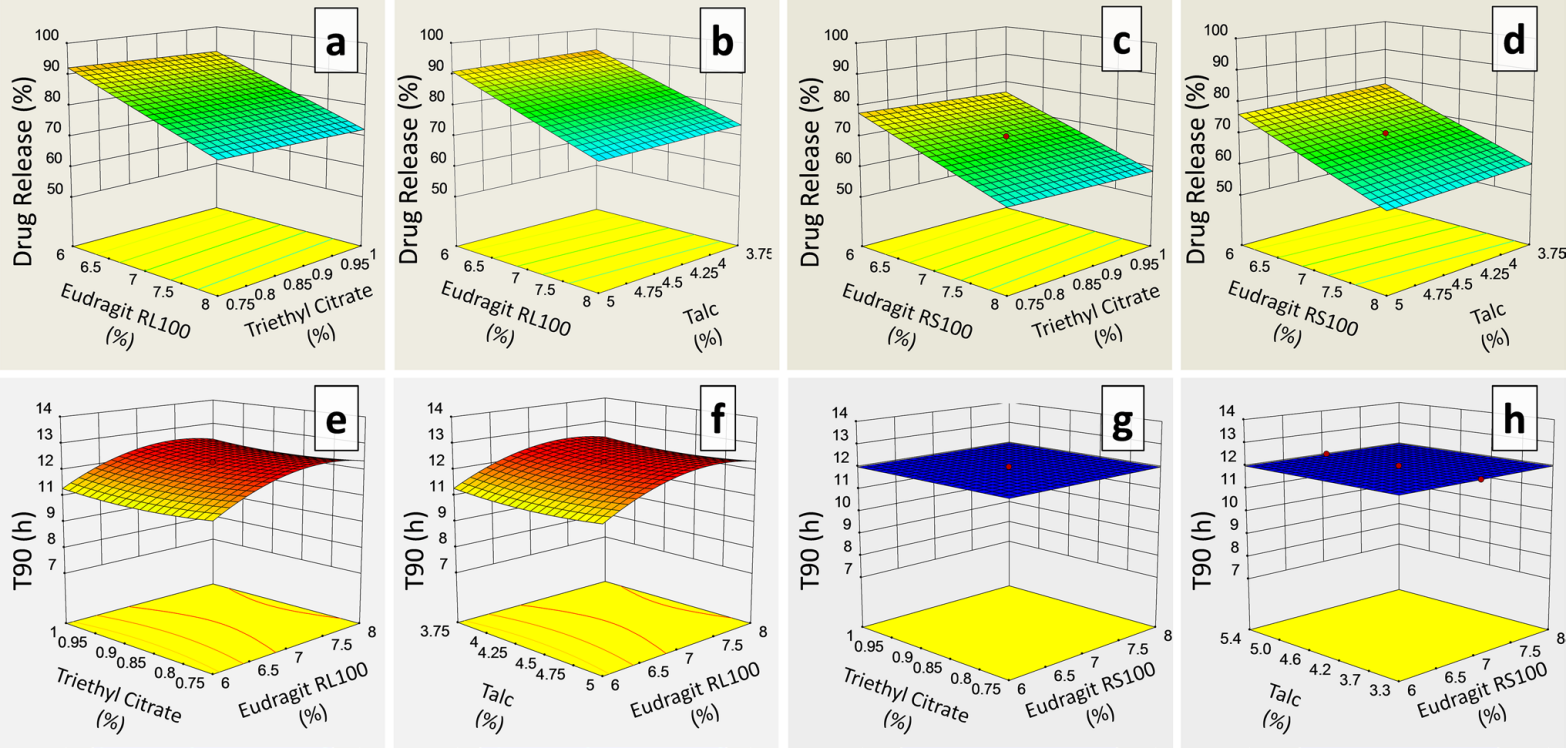
RSM plots of drug release and T90 as a function of different levels of (**a**, **b**, **e** & **f**) Eudragit RL100, TEC and Talc, and (**c**, **d**, **g** & **h**) Eudragit RS100, TEC and Talc.

Different concentrations 5.318–8.682% of RL100 and RS100 were coated on formulations FC11–FC30 and FC31–FC50, respectively. Stereo images of Eudragit RL100 and RS100 coated pellets are shown in Figs. 2a and 3a, respectively. Formulation FC11 coated with 5.318% RL100 released 98% drug within 8 h. Meclizine HCl release was effectively extended (94%) up to 12 h by formulations coated with 6% RL100 (FC12–FC15). However, pellets coated with 7% (FC16–FC25), 8% (FC26–FC29) and 8.682% (FC30) RL100 excessively sustained release of drug and at the end of 12 h only 79.971–81.915%, 72.799–74.997% and 65.448% drug was released respectively (Fig. 2b). Eudragit RS100 coating provided a more controlling effect on the release of Meclizine HCl as compared to RL100, even 5.318% RS100 coated pellets released only 84.585% drug at 12 h (Fig. 3b). It is evident from the RSM plots of RL100 (Fig. 4a) and RS100 (Fig. 4c) that drug release decreased with increased concentration of coated polymers. For RL100 time for 90%, drug release was increased with higher polymer load (Fig. 4e) but in the case of RS100, T90 was more than 12 h for all coated formulations showing no variation in the plot, illustrated in RSM plot (Fig. 4g). Model summary statistics of Eudragit RL100 and RS100 are shown in Table 2.

**Table 2:** Model summary statistics of drug release, T90, aspect ratio and eR of ER coated pellets of Meclizine HCl.

Models	Eudragit RL100^®^								Eudragit RS100^®^							
	Mean squares	F value	p value prob > F	SD	Adj. R-Sq	Pred. R-Sq	Press	Pred. Model	Mean Square	F value	p value prob > F	SD	Adj. R-Sq	Pred. R-Sq	Press	Pred. model
**Drug release (DR)**																
Linear	1.55	386.31	˂ 0.0001	1.030	0.984	0.975	30.950	Linear < 0.0001 Sig	7.46	72.73	˂ 0.0001	2.260	0.9189	0.886	136.160	Linear < 0.0001 Sig
2FI	1.97	0.35	0.7866	1.100	0.982	0.941	73.370		10.21	0.017	0.9968	2.510	0.9005	0.683	380.220	
Quadratic	2.43	0.99	0.4381	1.100	0.981	0.927	91.980		8.16	3.34	0.0640	2.020	0.9355	0.743	308.490	
Cubic	10.92	0.17	0.9452	1.350	0.972	− 0.921	2,406.86		27.33	0.74	0.5985	2.130	0.9279	− 4.017	6,023.49	
**Time for 90% drug release (T90)**																
Linear	1.69	1.49	0.2559	1.080	0.071	− 0.415	33.610	Quadratic 0.2438 Not Sig	0.00	–	–	–	–	–	–	N/A
2FI	2.32	0.00	1.0000	1.200	− 0.143	− 1.314	54.970		0.00	–	–	–	–	–	–	
Quadratic	1.96	2.97	0.0834	0.990	0.214	− 2.135	74.460		0.00	–	–	–	–	–	–	
Cubic	2.50	4.40	0.0531	0.640	0.667	− 22.158	550.01		0.00	–	–	–	–	–	–	
**Aspect ratio (AR)**																
Linear	6.685E − 004	5.46	0.0089	0.021	0.413	0.193	0.012	Linear 0.0065 Sig	1.239E − 004	0.54	0.6590	9.231E − 003	− 0.077	− 0.514	2.274E − 003	Quadratic 0.0121 Not Sig
2FI	9.191E − 004	0.000	1.0000	0.024	0.278	− 1.134	0.032		1.704E − 004	0.00	1.0000	0.010	− 0.326	− 1.787	4.188E − 003	
Quadratic	4.955E − 004	6.56	0.0100	0.016	0.684	− 0.257	0.019		5.760E − 005	12.45	0.0010	5.367E − 003	0.636	− 0.453	2.184E − 003	
Cubic	2.355E − 003	0.078	0.9863	0.020	0.499	− 33.883	0.520		7.664E − 005	4.14	0.0603	3.574E − 003	0.838	− 10.243	0.017	
**Two dimensional shape factor (eR)**																
Linear	6.833E − 004	56.69	˂0.0001	0.022	0.898	0.844	0.014	Quadratic < 0.0001 Sig	2.581E − 004	22.63	˂0.0001	0.013	0.773	0.710	4.309E − 003	Quadratic < 0.0001 Sig
2FI	8.858E − 004	0.26	0.8509	0.023	0.881	0.837	0.014		3.036E − 004	0.73	0.5515	0.014	0.761	0.433	8.434E − 003	
Quadratic	8.890E − 005	49.81	˂ 0.0001	6.667E − 003	0.990	0.959	3.501E − 003		9.224E − 005	14.22	0.0006	6.791E − 003	0.941	0.758	3.596E − 003	
Cubic	2.867E − 004	0.83	0.5539	6.912E − 003	0.989	0.277	0.063		6.860E − 005	8.58	0.0117	3.381E − 003	0.985	− 0.016	0.015	

To determine the influence of plasticizer and anti-tacking agent on Meclizine HCl release and T90, Eudragit RL100 (FC11–FC30) and RS100 (FC31–FC50) formulations were prepared with a variable amount of TEC (0.665–1.085) and talc (3.324–5.426). The release of Meclizine HCl was not significantly controlled by varying amounts of TEC and talc which is indicated in RSM plots, Fig. 4a,b for RL100 and Fig. 4c,d for RS100 respectively. T90 also remained unaffected by increased concentration of TEC and talc in both Eudragit RL100 (Fig. 4e,f) and RS100 (Fig. 4g,h) coated pellets.

#### Sphericity of Eudragit coated pellets

**Figure 5:**
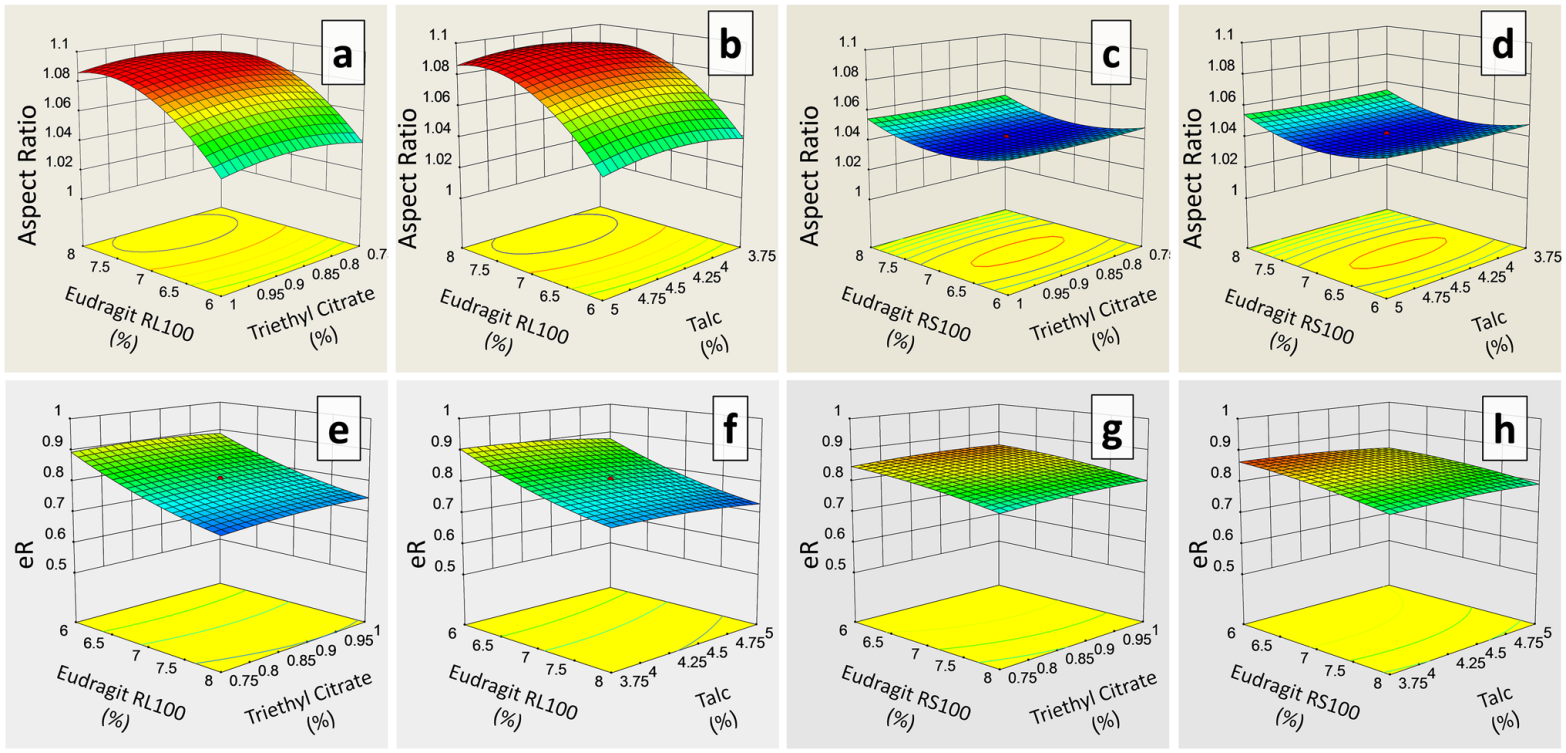
RSM plots of aspect ratio and eR as a function of different levels of (**a**, **b**, **e** & **f**) Eudragit RL100, TEC and Talc, and (**c**, **d**, **g** & **h**) Eudragit RS100, TEC and Talc.

The influence of coated polymers on the sphericity of pellets was also evaluated. Different shape parameters like area, perimeter, circularity, feret diameter, roundness, projection sphericity, dce, aspect ratio, and two-dimensional shape factors were determined (Supplementary Table S1). Area and perimeter of Eudragit RL100 coated pellets were ranged from 23,865 to 28,065 and 588.907–621.555, respectively, whereas, for RS100 these were ranged from 26,003 to 28,948 and 588.190–623.716, respectively. Circularity, feret diameter and projection sphericity of Eudragit RL100 coated pellets were ranged from 0.858 to 0.965, 180.945–198.789, 0.851–0.932, respectively, whereas, for RS100 these were ranged from 0.885 to 0.966, 187.547–197.085, 0.813–0.892, respectively. For Eudragit RL100 coated pellets the circle equivalent diameter dce and elongation ratio ER were ranged from 174.360 to 189.081 and 0.958–1.000, respectively, and for RS100 these were ranged from 182.002 to 192.032 and 0.960–0.986, respectively. The aspect ratio and shape factor of Eudragit RL100 coated pellets were ranged from 1.018 to 1.085 and 0.714–0.975, respectively, whereas, for RS100 these were ranged from 1.042 to 1.066 and 0.758–0.873 respectively. Response surface plots of RL100 indicated that aspect ratio of Meclizine HCl coated pellets were increased with increasing concentration of coated polymer (Fig. 5a,b), but the influence of RS100 on aspect ratio was variable (Fig. 5c,d). Two-dimensional shape factor (eR) was decreased with an increased amount of both the coated polymers RL100 and RS100 (Fig. 5e–h). Although both aspect ratio and two-dimensional shape factor were within an acceptable limit. The ANOVA analysis of aspect ratio and eR of Eudragit RL100 coated pellet formulations suggested linear and quadratic models respectively, for data fitting (P < 0.05), while for RS100 quadratic model was suggested for both the responses. The fit summary of aspect ratio and eR is given in Table 2.

The aspect ratio and eR of Eudragit RL100 coated pellet formulations were independent of the amount of TEC as shown in Fig. 5a,e, respectively. Talc slightly affected eR of RL100 coated pellets, illustrated in Fig. 5f. However, aspect ratio and eR were slightly influenced by TEC (Fig. 5c,g) and talc (Fig. 5d,h) levels in RS100 coated pellets.

### Drug-excipient interactions

The characteristic peaks of Meclizine HCl at 2,986.61 cm^−1^ (C–H str.), 1658.57 cm^−1^ (C=C str.), 1,499.21 cm^−1^ (CH_2_ bending), 1,433.83 cm^−1^ (CH_3_), 1,270.37 cm^−1^ (C–N str.), 939.39 (C–H bending), 808.63 (C–H bending), 718.78 cm^−1^ (C–Cl str.) are illustrated in Supplementary Fig. S2a. FTIR spectra of IR core pellets FC9, ER coated pellets FC12 (RL100) and FC31 (RS100) formulations are shown in Supplementary Fig. S2b–d, respectively, indicating the absence of any drug-polymer interactions.

### Characterization of IR core and ER coated pellets

#### Moisture content, yield, flow properties, and friability

The moisture content of Meclizine HCl IR core pellets was found to be 20.993% ± 2.5. The yield of IR core pellets was enhanced in the presence of PVP up to 92%. The results of the angle of repose, compressibility index, and Hausner ratio suggested that coated pellets had excellent flow properties. Friability of all pellet formulations was adequate representing that pellets were strong enough to bear attrition and shock during transportation, consumption, and storage. The content (percent) of Meclizine HCl in each pellet formulation was within the label claim (60 mg/capsule).

#### Surface morphology

**Figure 6:**
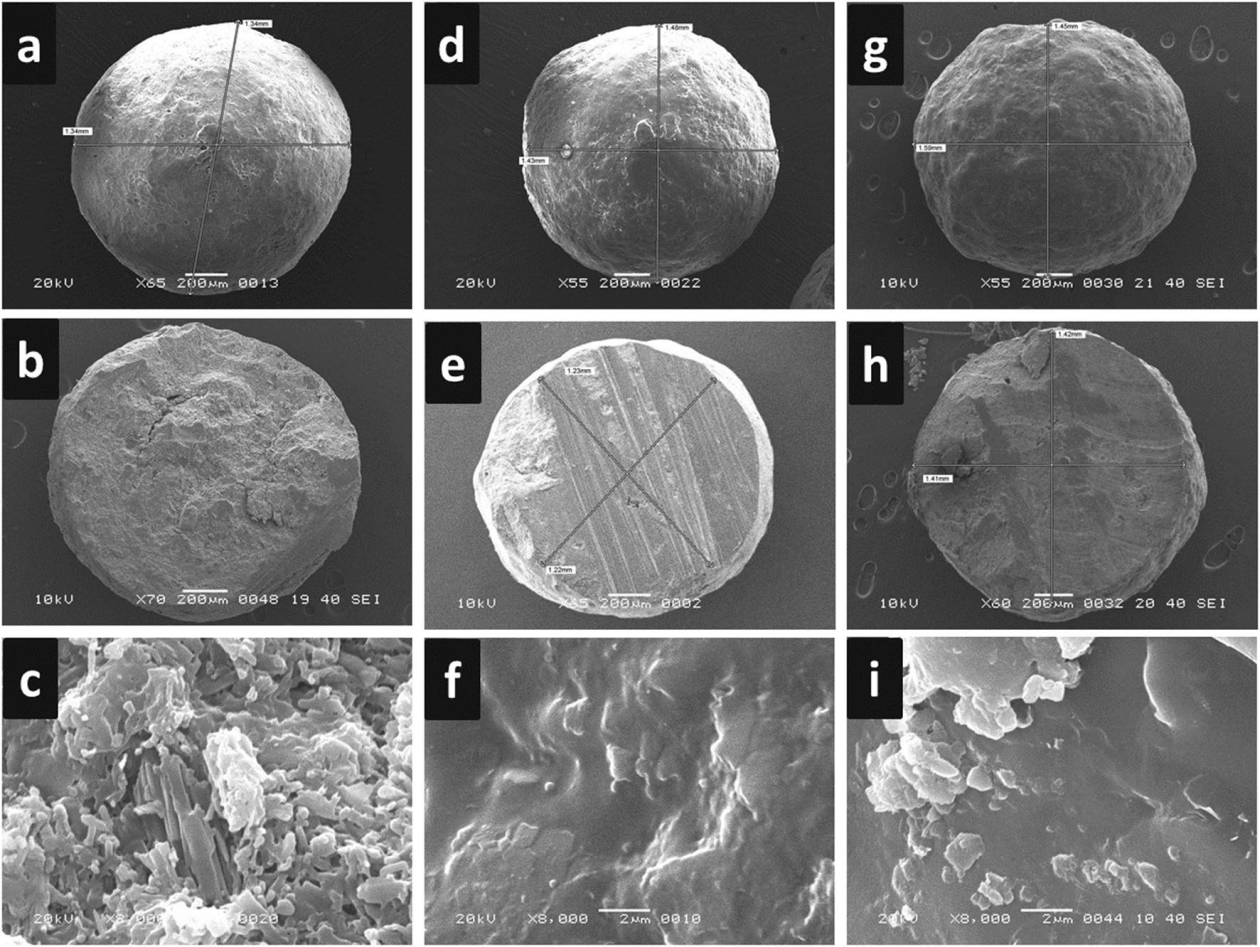
SEM images showing intact pellets and cross-sections of (**a**–**c**) IR drug core pellets (**d**–**f**) Eudragit RL100 coated pellets and (**g**–**i**) Eudragit RS100 coated pellets.

The surface morphology of the IR core and coated pellets was revealed by SEM. Meclizine HCl IR cores were appeared spherical and intact in shape (length × width = 1.34 mm) with numerous minute pores on the smooth surface as shown in Fig. 6a. The uniform MCC network without any pores and the uncoated edges of half piece of core pellets are shown in the cross-section of cores (Fig. 6b). The presence of numerous pores as seen in Fig. 6c zoomed at 5 µm. The external morphology of Eudragit coated pellets indicated that the coating was denser in the case of RS100 (Fig. 6g) as compared to RL100 (Fig. 6d). The length and the width of the cross-section of Eudragit RL100 and RS100 coated pellets further confirmed the formation of spherical Meclizine HCl IR cores, shown in Fig. 6e,h, respectively. The application of thin films of Eudragit RL100 and RS100 reduced the number of pores present on the surface of IR Meclizine HCl cores visualized at 5 µm as shown in Fig. 6f,i, respectively.

#### The thickness of coated polymeric layers

**Figure 7:**
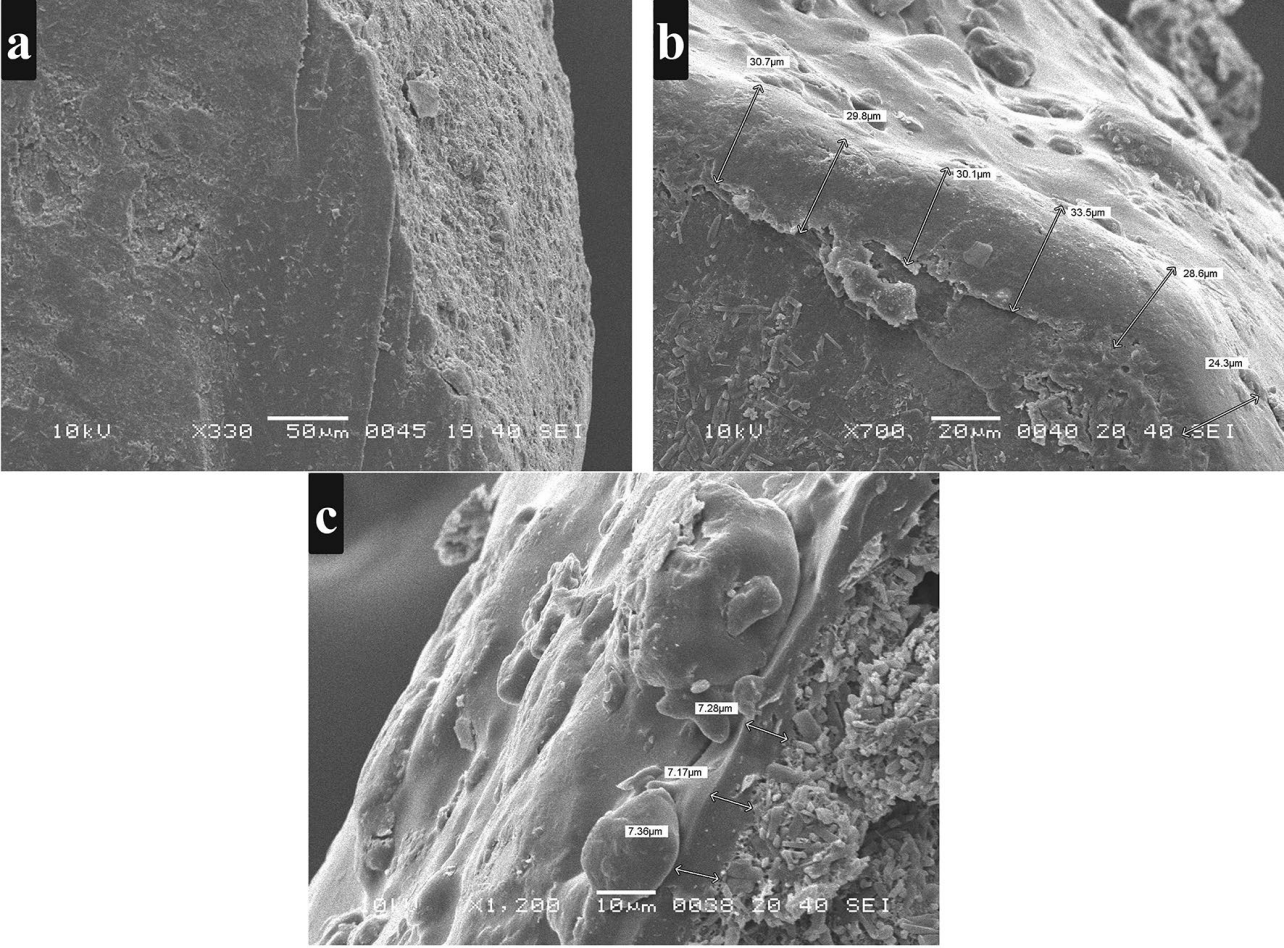
SEM images showing edges and thickness of polymeric layer of (**a**) IR drug core pellets (**b**) Eudragit RL100 coated pellets (**c**) Eudragit RS100 coated pellets.

The thickness of Eudragit RL100 and RS100 film coats was measured using SEM. In comparison to uncoated pellets (Fig. 7a), the formation of thick polymeric films/layers on the surrounding of coated pellets were evident in Fig. 7b,c for Eudragit RL100 and RS100 coats respectively. The thickness of the polymeric layers was measured at various cross-sectional points of the pellets.

#### Energy dispersive spectroscopy (EDS)

Elements carbon, oxygen, and chlorine were found on the surface of IR core pellets of Meclizine HCl (Supplementary Fig. S3a). The highest content of carbon was due to an organic drug-containing a carbon chain as previously reported for matrix ER pellets^1^. Similar elements carbon, oxygen, and chlorine were also found on the surface of Eudragit RL100 and RS100 coated pellets as shown in Supplementary Fig. S3b,c, respectively. Besides these three elements, Eudragit RL100 coated pellets showed additional peaks of magnesium, silicon, iron, and copper at 5 µm (Supplementary Fig. S3b) whereas, magnesium, silicon, aluminum, and copper were also found in RS100 coated pellets (Supplementary Fig. S3c).

### Influence of pH on drug release from the core and coated pellets

Meclizine HCl IR cores were evaluated in different dissolution media and pronounced pH-dependent drug release was observed, shown in Fig. 1b. Meclizine HCl is an acidic salt of a weakly basic drug having pKa 6.12. Therefore, its solubility and ionization are reduced at alkaline pH and increased at acidic pH. This may be due to the conversion of the hydrochloride salt to its less soluble free base^1^. Meclizine HCl release was decreased as the pH was increased from 1.2 to 6.8. The optimized Eudragit RL100 coated formulations were also evaluated for pH effect. The optimized formulations (FC12–FC15) released more than 90% Meclizine HCl within 12 h at pH 1.2. When the pH was increased to 4.5, drug release was reduced to 50% at the end of 12 h. Less than 45% drug was released from Eudragit RL100 coated pellet formulations at pH 6.8. Thus, Eudragit coated pellets exhibited the same pH-dependent behavior as that of IR drug cores i.e. faster in an acidic environment than an alkaline one. A similar pH-dependent drug release from Eudragit based formulations was also observed by other researchers^35,36^. On the contrary, pH-independent drug release from Eudragit formulations was also reported^33,37^ claiming ionization of quaternary ammonium groups occurred at all pH levels of the gastrointestinal tract.

### Drug release kinetics

#### Model-dependent methods

Based on best goodness of fit, the most appropriate model was selected for all formulations. Kinetics of Meclizine HCl pellets coated with 5.3% (FC11) and 6% (FC12–FC15) Eudragit RL100 were best explained by Korsmeyer–Peppas (R^2^ = 0.9892–0.9941) followed with Zero-order (R^2^ = 0.9787–0.9879) and Higuchi (R^2^ = 0.9770–0.9882) models. When the concentration of coated polymer RL100 was increased to 7% (FC16–FC25) Hixson–Crowell (R^2^ = 0.9856–0.9933) and Zero order (R^2^ = 0.9747–0.9885) models were applied. Zero-order model (0.9594–0.9897) was only obeyed at 8% (FC26–FC29) and 8.6% (FC30) Eudragit RL100 coating levels (Supplementary Table S2).

Korsmeyer–Peppas model (R^2^ = 0.9936–0.9978) showed the highest linearity when Meclizine HCl cores were coated with 5.3% (FC31), 6% (FC32–35) and 7% (FC36–FC45) Eudragit RS100. Hixson–Crowell (R^2^ = 0.9730–0.9965), Higuchi (R^2^ = 0.9730–0.9890) and Zero-order (R^2^ = 0.9796–0.9967) models also indicated applicability at these concentrations of RS100. When the concentration of coated polymer RS100 was increased to 8% (FC46–FC49) and 8.6% (FC50) Zero-order model (R^2^ = 0.9727–0.9892) was applied. The release constant (*k*) of all kinetic models was progressively reduced with increasing concentration of Eudragit coated polymers. The MDT of Eudragit RL100 and RS100 coated pellets was ranged from 3.27 to 6.141 h and 5.138–5.708 h respectively (Supplementary Table S3). The highest dissolution efficiency (31.904%) was observed in the case of Eudragit RL100 coated pellets.

#### Model-independent method

Out of forty (FC11–FC50) coated pellet formulations, FC12, coated with 6% Eudragit RL100, 0.75% TEC and 3.75% talc was selected as a reference encapsulated pellet formulation. Among different coated formulations, dissolution profile of FC12 was similar with FC13–FC25 (coated with 6% and 7% RL100) and FC31 (coated with 5% RS100), having *f*_2_ = 89.167, 84.927, 82.445, 59.961, 59.961, 59.961, 59.961, 59.961, 59.961, 58.611, 55.606, 55.606, 60.781 and 67.885, respectively.

### Stability

Out of forty (FC11–FC50) ER pellet formulations, only four formulations (FC12–FC15), coated with 6% Eudragit RL100, released more than 90% drug within 12 h. These formulations were considered as optimized ER pellet formulations. The optimized formulations showed acceptable stability for 6-months at 40 °C ± 2 °C/75% ± 5% RH. No significant difference in physical appearance, in vitro drug release and drug content was observed. Results indicated shelf-life of 45 months with a 90% lower acceptance limit of label claim^25^.

### Development and validation of the bioanalytical method

#### Development of the bioanalytical method

According to FDA and ICH guidelines, a new high performance liquid chromatographic method with fluorescence detection was developed and validated for the determination of Meclizine in human plasma^15,16^. The composition of the mobile phase was adopted from the official monograph of Meclizine HCl assay^20^. Mobile phase composed of 1-heptane sulfonate buffer and ACN in a ratio of 300:700 at pH 3 resolved plasma phospholipids and proteins from drug and IS peaks. A previous study, conducted for the simultaneous determination of Meclizine HCl with five other drugs, reported 10 min retention time of Meclizine HCl, when sodium salt of heptane sulphonic acid (0.01 M) and acetonitrile in a ratio of 42:58% v/v adjusted to pH 3 was used with PDA detector^10^.

Four different extraction procedures liquid extraction, liquid extraction in two steps, double extraction, and protein precipitation were evaluated to achieve the maximum clean-up of the sample. Liquid–liquid extraction in a single (Supplementary Fig. S4a,b) and two steps (Supplementary Fig. S4c,d) failed to provide acceptable sample clean-up, whereas, double extraction showed insignificant analytical recovery of Meclizine (Supplementary Fig. S4e,f). Protein precipitation using acetonitrile as a deproteinating agent with heat treatment for 5 min (Supplementary Fig. S4g,h) and 10 min (Supplementary Fig. S4i,j) indicated multiple plasma peaks with broad analyte peak. Protein precipitation using an altered ratio of a deproteinating agent at 1:0.9 (Supplementary Fig. S4k,l) and 1:0.8 yielded the desirable and consistent recovery of the analyte with acceptable peak shape. Other extraction solvents previously reported for Meclizine were chloroform^7^, perchloric acid^8^, acetonitrile^9^, and 50% methanol^12^. The solid-phase extraction method has also been used for sample pre-treatment^10^. For the selection of internal standards, the structural analogues Flunarizine and Cinnarizine were preferred^38^. But the co-elution of these compounds with Meclizine rendered them unfit for use, even after monitoring these compounds at their respective wavelengths on different acquisition channels. (Supplementary Fig. S5a,b). Another reported compound was Pyridoxine^10^ which failed to show any peak at the used fluorescence wavelengths (Supplementary Fig. S5c). Hence, it was expected that a strongly fluorescent drug can be considered as an internal standard which exhibits satisfactory response even with low analytical recovery. Therefore, quinolone antibiotics Pefloxacin^39^, Ofloxacin^40^, and Levofloxacin^41^ were tried. The retention time of all the three quinolones was between 2.5 and 2.6 min with satisfactory recovery but the peak shape of Levofloxacin and Pefloxacin was unacceptable (Supplementary Fig. S5d,e, respectively) and thus, excluded from further investigation. While Ofloxacin exhibited a sharp peak at 285–460 nm (Channel 2) as illustrated in Supplementary Fig. S5f which led to the use of Ofloxacin as an internal standard for Meclizine.

#### Validation of the bioanalytical method

**Figure 8:**
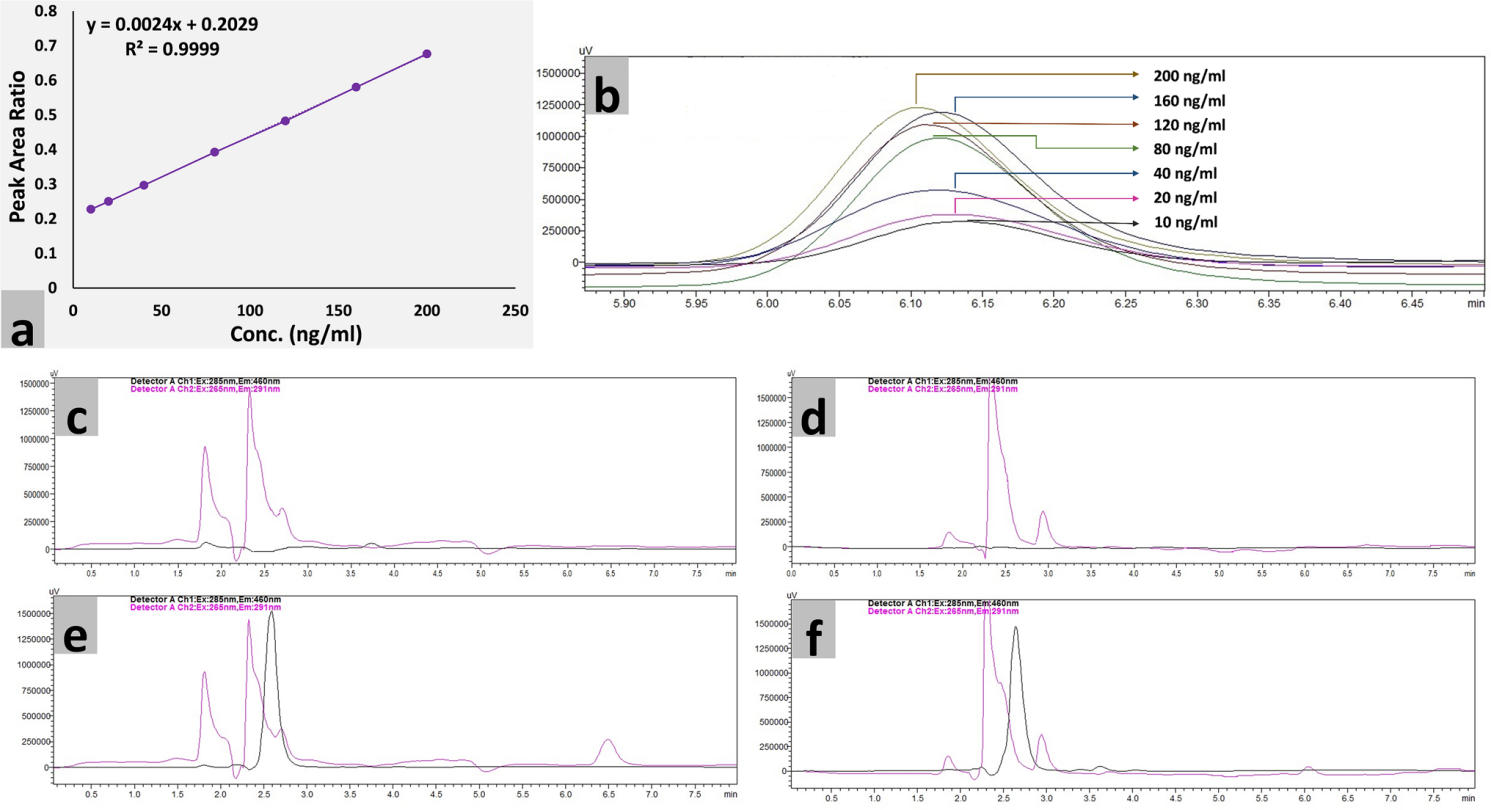
Validation of newly developed fluorescent method for quantification of Meclizine in terms of (**a**) calibration curve (**b**) linearity (**c** & **d**) selectivity (**d**) LLOQ and (**e**) LLOD.

The newly developed HPLC method with fluorescence detection was validated according to FDA and ICH guidelines which include accuracy, precision, selectivity, sensitivity, reproducibility, robustness, and stability^15,16^. No interfering peak at the analyte retention time (6.2 min) indicated selectivity of the method in the presence of plasma components, shown in Fig. 8c,d. The linearity was established within a concentration range of 10–200 ng/ml, shown in Fig. 8a,b. The accuracy in terms of deviation (%) from the nominal concentrations was ranged from 99.9 to 101.7% (Supplementary Table S4). Less variability in retention time and the insignificant difference in recovery mean due to deliberately varied chromatographic parameters indicated robustness of the newly developed method (Supplementary Table S5). The lower limit of quantification (LLOQ) and lower limit of detection (LLOD) was found to be 10 ng/ml and 1 ng/ml, respectively, shown in Fig. 8e,f. Consistent analytical recovery was obtained over the calibration curve as shown in Table 3. Results of freeze and thaw stability, long-term stability, stock solution stability, benchtop, and autosampler stability and their gross mean degradation are also indicated in Table 3.

**Table 3:** Recovery and stability of Meclizine under different conditions.

Validation parameters	Freeze–Thaw Stability		Long term stability		Stock solution stability	
	Low Conc. (10 ng/ml)	High Conc. (200 ng/ml)	Low Conc. (10 ng/ml)	High Conc. (200 ng/ml)	Low Conc. (10 ng/ml)	High Conc. (200 ng/ml)
	FT Cycle 3	FT Cycle 3	After 6 weeks	After 6 weeks	After 6 weeks	After 6 weeks
Mean ± SD	9.880 ± 0.1378	196.888 ± 1.757	9.775 ± 0.268	195.806 ± 0.967	10.008 ± 0.050	199.975 ± 1.032
Precision (CV) %	1.395	0.893	2.739	0.494	0.501	0.516
Accuracy (%)	98.798	98.404	97.746	97.903	100.084	99.987
Gross mean degradation (%)	1.119	0.975	1.300	1.429	0.237	0.311

Validation parameters	Benchtop stability 24 h		Autosampler stability 65 h		Recovery	
	Low Conc. (10 ng/ml)	High Conc. (200 ng/ml)	Low Conc. (10 ng/ml)	High Conc. (200 ng/ml)	Low Conc. (10 ng/ml)	High Conc. (200 ng/ml)
Mean ± SD	10.016 ± 0.294	200.109 ± 0.651	10.006 ± 0.638	199.899 ± 0.519	Peak ratio of drug and IS	Peak ratio of drug and IS
Precision (CV) (%)	0.965	0.487	0.519	0.742	0.282 (MP)	0.674 (MP)
Accuracy (%)	100.160	100.054	100.060	99.949	0.279 (Plasma)	0.658 (Plasma)
Gross mean degradation (%)	0.018	0.571	0.028	0.781	98.227%	99.822%

### Pharmacokinetics of Meclizine HCl pellet formulations

Among different coated formulations, FC12, coated with 6% Eudragit RL100, 0.75% TEC, and 3.75% talc was selected for pharmacokinetic study due to the formation of highly spherical and smooth surface pellets having zero-order release kinetics. Although, formulations FC13–FC15 also showed similar properties but the least quantities of ingredients were used in FC12. Pharmacokinetics of the newly developed and optimized Meclizine HCl ER pellet formulation (FC12) was determined in healthy adult volunteers and out of thirty subjects, twenty-four participated and completed the study. Each part comprised of 12 volunteers (n = 12). Since Meclizine HCl is not available in ER dosage form, comparative pharmacokinetic of newly developed ER Meclizine HCl pellets (60 mg) was carried out with IR core pellets of Meclizine HCl having the same dose (60 mg). Both IR and ER encapsulated pellet formulations were well tolerated by all the volunteers without any adverse effects. Several studies reported comparative pharmacokinetic and bioavailability of a single dose ER and IR formulations^42,43^ (Table 4).

**Table 4:** Pharmacokinetic parameters of single dose Meclizine HCl IR and ER pellets (Part 1) and ER pellets under fasted and fed conditions (Part 2).

Parameters	Part 1 Study (n = 12)		Part 2 Study (n = 12)	
	IR	ER	ER (Fasted)	ER (Fed)
Cmax (ng/ml)	98.051 ± 0.020	84.052 ± 0.020	84.033 ± 0.030	80.049 ± 0.031
Tmax (h)	3.029 ± 0.0007	5.116 ± 0.002	5.117 ± 0.001	5.117 ± 0.002
AUC_0–t_ (ng/ml h)	1,169.964 ± 0.565	1,181.226 ± 0.518	1,181.228 ± 0.628	1,193.615 ± 0.425
AUC_0–∞_ (ng/ml h)	1,253.417 ± 0.593	1,265.117 ± 0.587	1,265.383 ± 0.933	1,275.111 ± 0.511
MRT_0–t_ (h)	14.795 ± 0.008	14.652 ± 0.009	14.652 ± 0.011	14.727 ± 0.004
MRT_0–∞_ (h)	17.561 ± 0.008	17.418 ± 0.010	17.427 ± 0.020	17.374 ± 0.007
Cl (l/h)	47.869 ± 0.023	47.426 ± 0.022	47.417 ± 0.035	47.055 ± 0.019
Vz (l)	399.010 ± 0.290	397.022 ± 0.823	397.463 ± 0.889	383.293 ± 0.599
Kel (h^−1^)	0.127 ± 0.000	0.119 ± 0.000	0.119 ± 0.000	0.123 ± 0.000
T_1/2Kel_ (h)	5.448 ± 0.009	5.803 ± 0.011	5.810 ± 0.0150	5.646 ± 0.009

#### Part 1: Single dose IR (60 mg) vs. ER (60 mg) pellets study

**Figure 9:**
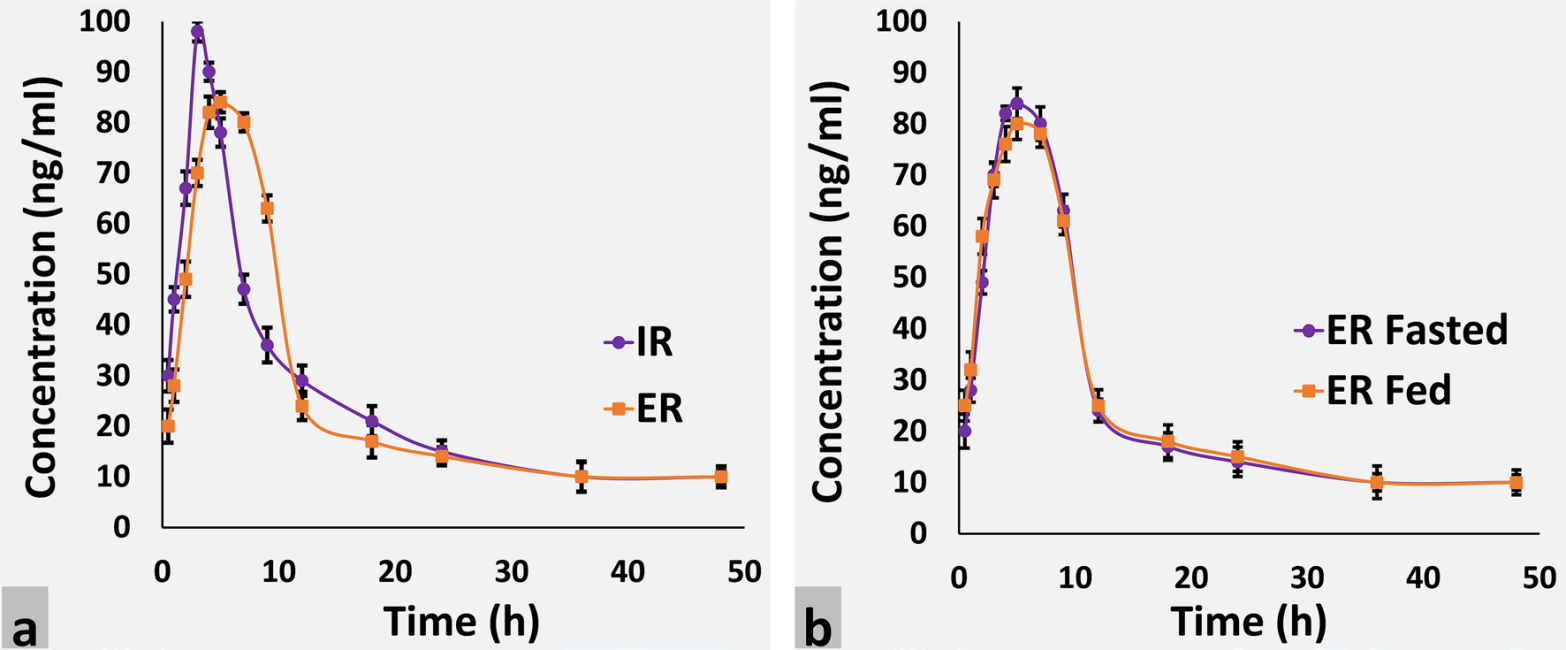
Mean plasma concentration versus time profile comparison of (**a**) IR Meclizne HCl pellets (60 mg) and ER Meclizine HCl pellets (60 mg) (**b**) ER Meclizine HCl pellets under fasted and fed conditions.

The details of volunteers (n = 12) participated in study I (single dose IR vs. ER pellet formulations) are indicated in Supplementary Table S6. Comparative plasma concentration–time profile of IR (60 mg) and ER (60 mg) pellet formulations of Meclizine HCl is illustrated in Fig. 9a. Pharmacokinetic parameters of IR and ER encapsulated pellet formulations are given in Table 4. The mean C_max_ of Meclizine HCl IR encapsulated pellet formulation (60 mg) was 98.051 ng/ml and ER encapsulated pellet formulation was 84.052 ng/ml under the fasted state. The T_max_ of the IR encapsulated pellet formulation was 3.029 h and ER encapsulated pellet formulation was 5.116 h. The mean AUC_0–t_ and mean AUC_0–∞_ were 1,169.964 ng/ml × h and 1,253.417 ng/ml × h for IR pellets and 1,181.226 ng/ml × h and 1,265.117 ^+^ng/ml respectively for ER pellets under fasted condition. The mean MRT_0–t,_ MRT_0–∞,_ clearance (Cl), and volume of distribution (Vz) are given in Table 4. Wang et al. determined the pharmacokinetics of Meclizine HCl 25 mg tablets in 20 healthy volunteers and reported C_max_ 80.1 + 51.9 ng/ml, T_max_ 3.1 + 1.4 h, AUC_0–24_ 544.3 + 511.6 h ng/ml and AUC_0_ 566.54 + 534.75 h ng/ml, Vz 6.78 + 3.52 l/kg, Cl 0.14 + 0.02 l/h/kg and the terminal elimination half-life 5.21 + 0.8 h^12^. In another study, Wang et al., prepared Meclizine oral solution (MOS) and reported its C_max_ 99.43 + 48.34 ng/ml, T_max_ 1.28 + 0.74 h, AUC_0-24_ 542 + 410.53 ng/ml × h, AUC_0_ 564.03 + 439.96 ng/ml × h, 40 + 3.29 l/kg Vz, 0.14 + 0.02 l/h/kg Cl and 5.24 + 0.82 h T_1/2_^2^. Kharshoum and Aboutaleb prepared Meclozine HCl microspheres using HPMC and compared its pharmacokinetics with marketed tablet Dramamine (25 mg). They reported C_max_ 61.29 + 6.01 ng/ml and 70.34 + 2.265 ng/ml, T_max_ 4.66 + 0.94 h and 2.17 + 0.48 h, AUC_0–t_ 787.25 + 89.14 ng/ml × h and 572.37 + 68.34 ng/ml × h and AUC_0_ 870.2867 + 97.23 ng/ml × h and 594.3509 + 73.14 ng/ml × h, MRT 10.9479 + 1.14 h, 7.7589 + 1.13 h and half-life 6.5438 + 0.64 h and 4.8833 + 0.51 h for Meclizine HCl microspheres and Dramamine tablets respectively^13^. Sher et al. developed the HPLC method for the simultaneous determination of Meclizine in formulations and human serum and also determined the pharmacokinetic application of the newly developed method. The pharmacokinetic study was conducted in 24 healthy male Pakistani volunteers and C_max_, T_max_, AUC, and half-life were found to be 0.42 + 0.36 µg/ml, 3.5 + 0.40 h, 5.10 + 2.52 h µg/ml and 5.10 + 2.52 h, respectively^10^.

#### Part 2: Single dose ER (60 mg) food effect study

The details of volunteers (n=12) participated in study 2 (single dose ER food effect) are indicated in Supplementary Table S6. Comparative plasma concentration–time profile of ER (fasted) and ER (fed) pellet formulations of Meclizine HCl is illustrated in Fig. 9b and pharmacokinetic parameters of ER fasted and ER fed encapsulated pellet formulations are given in Table 4. The mean C_max_ of Meclizine HCl ER encapsulated pellet formulation (60 mg) under a fasted state was 84.033 ng/ml and under a fed state was 80.049 ng/ml. The mean T_max_ of ER encapsulated pellet formulation under both fasted and fed conditions was 5.117 h. The mean AUC_0–t_ and the mean AUC_0–∞_ were 1,181.228 ng/ml × h and 1,265.383 ng/ml × h under the fasted state for ER pellets and 1,193.615 ^+^hng/ml and 1,275.111 ^+^hng/ml, respectively under the fed state. The mean MRT_0–t_ and MRT_0–∞_ were recorded as 14.652 h and 17.427 h under fasted state and 14.727 h and 17.374 h respectively under the fed state for ER Meclizine HCl pellet formulations. In the fasted state, the volume of distribution (Vz) and clearance (Cl) of ER Meclizine HCl pellets were 397.463 l and 47.417 l/h whereas, 383.293 l Cl and 47.055 l/h Vz respectively were observed under fed conditions. The elimination rate constant (Kel) and its respective half-life of IR and ER Meclizine HCl pellet formulations in fasted condition and ER pellets under fasted and fed conditions were also determined (Table 4).

### Statistical analysis for establishing bioequivalence

#### Part 1: single-dose IR (60 mg) vs. ER (60 mg) pellet study

The mean C_max_ of IR reference pellet formulation was 98.0512 ng/ml and for ER test pellet formulation was 84.0517 ng/ml. The GMR (Geometric mean ratio) of ER/IR for C_max_ values was 0.8572 and associated 90% CI was 0.8571–0.8573 after a single dose which was closed to the boundaries of the bioequivalence statistical limits (0.85–1.25). The mean T_max_ of IR reference pellet formulation was 3.0295 h and ER test pellet formulation was 5.1163 h. The GMR (Geometric mean ratio) for T_max_ values was 1.6888 with a 90% CI (1.6884–1.6893). The mean AUC_0-t_ of IR reference pellet formulation and ER test pellet formulation were 1,169.9639 ng/ml × h and 1,181.2261 ng/ml × h respectively, under fasted condition. The GMR was 1.0096 with a 90% CI (1.0093–1.0100). Similarly, the mean AUC_0_ of IR reference pellet formulation and ER test pellet formulation were found to be 1,253.4172 ng/ml × h and 1,265.1170 ng/ml × h respectively with GMR 1.0093. The 90% CI limit was 1.0090–1.0096. The GMR of elimination rate constants Kel at 90% CI was noted to be 0.9410 (0.9382–0.9437) for IR reference pellets and ER test pellets under fasted condition. The effect of period, sequence, and subject was recorded as not significant. The GMR (1.0043) of elimination half-life, T_1/2Kel_ (1.0033–1.0053) was noted with 90% CI under the fasted state for IR Meclizine HCl reference pellet formulation, and ER test pellet formulation (Table 5).

**Table 5:** Bioequivalence of single dose Meclizine HCl (60 mg) IR and ER pellets (Part 1) and ER pellets under fasted and fed conditions (Part 2) with geometric mean ratios at 90% CI (log-transformed).

Parameters	Part 1 Study (n = 12)		Part 2 Study (n = 12)	
	ER/IR ratio	90% CI	ER (Fed)/fasted ratio	90% CI
Cmax (ng/ml)	0.8572	0.8571–0.8573	0.9526	0.9524–0.9528
Tmax (h)	1.6888	1.6884–1.6893	1.0903	1.0900–1.0907
AUC_0−t_ (ng/mL h)	1.0096	1.0093–1.0100	1.0105	1.0102–1.0108
AUC_∞_ (ng/mL h)	1.0093	1.0090–1.0096	1.0077	1.0073–1.0081
Kel (h^−1^)	0.9410	0.9382–0.9437	1.0300	1.0269–1.0331
T_1/2Kel_ (h)	1.0043	1.0033–1.0053	0.9718	0.9701–0.9735

#### Part 2: Single dose ER (60 mg) food effect study

The mean C_max_ of ER fasted reference pellet formulation was 84.0328 ng/ml and for ER fed test pellet formulation was 80.0492 ng/ml. The GMR (Geometric mean ratio) of fed/fasted for C_max_ values was 0.9526 and associated 90% CI was 0.9524–0.9528 after a single dose. The mean T_max_ of ER fasted reference pellet formulation was 5.117 h and for ER fed test pellet formulation was 5.5798 h. The GMR (Geometric mean ratio) for T_max_ values was 1.0903 with a 90% CI (1.0900–1.0907). The mean AUC_0-t_ of ER fasted reference pellet formulation and ER fed test pellet formulation were 1,181.2283 ng/ml × h and 1,193.6146 ng/ml × h, respectively. The GMR was 1.0105 with 90% CI (1.0102–1.0108). Similarly, the mean AUC_0_ of ER reference pellet formulation and ER test pellet formulation were found to be 1,265.3826 ng/ml × h and 1,275.1107 ng/ml × h respectively, with GMR 1.0077. The 90% CI limit was 1.0073–1.0081 (Table 5). The GMR of elimination rate constant Kel at 90% CI was noted to be 1.0300 (1.0269–1.0331) for ER reference pellets under fasted and ER test pellets under fed conditions. The effect of the period, sequence, and subject were recorded as not significant. The GMR of 0.9718 elimination half-life, T_1/2Kel_ (0.9701–0.9735) was noted with 90% CI for ER Meclizine HCl reference pellet formulation under fasted and ER test pellet formulation under fed state.

Drug absorption is mainly influenced by the food intake depending on the drug physicochemical properties and GIT physiological changes. Food affects the (1) C_max_ and T_max_ by reducing the gastric emptying, (2) solubility, dissolution and bioavailability of weak acids and bases by changing the gastric pH, (3) absorption from the small intestine by increasing viscosity of the intestinal contents which slows intestinal membrane perfusion, (4) digestion and emulsification of the drugs by increasing secretion of bile into the intestine^44,45^. The absorption and bioavailability of BCS class II drugs (low solubility and high permeability) are increased with concomitant administration of food. Maximum time is available for solubilization of the drug as food enhances the stomach residence time. Moreover, food increases the production of the phospholipids, bile salts, and digestive products which in turn promotes the solubilization of drugs^46^. In addition to the presence of food, the type of dosage form also affects gastric emptying. Pellets and solutions were emptied rapidly from the stomach during the digestive mood, whereas emptying of single units was delayed even in the presence of light breakfast^47^. The effect of food on Meclizine HCl was found insignificant because ER formulation was designed in the form of pellets which were emptied rapidly from the stomach during digestive mood as well.

## Conclusion

IR drug core pellets prepared only with MCC and lactose were not adequately spherical, uniform, and firm. This was effectively tackled by incorporating PVP (2–5%). Adjustments in spheronization speed and time yielded the formulation bearing optimal properties. QbD analysis showed comparatively superior film-forming property of ammonio methacrylate copolymer type A (RL100) than type B (RS100), to attain adequate controlled release property within the desired time period (12 h). Thus, optimized encapsulated ER pellet formulation was achieved with 6% Eudragit RL100, 0.75% TEC, and 3.75% talc. Lower consumption of coated polymer was hence, achieved with zero-order kinetics. Furthermore, for the estimation of Meclizine in human plasma, a new, sensitive, simple, robust, and less time-consuming HPLC-fluorescence bio-analytical method was developed and validated. A comparative pharmacokinetic study was conducted between ER pellets (test) and IR pellets (reference) using a single dose of 60 mg Meclizine HCl. Both IR and ER pellet formulations were well tolerated and their pharmacokinetic parameters were comparable in terms of AUC_0–t_ and AUC_0–∞_ values. The C_max_ of IR pellets (98.051 ng/ml) was higher than the ER pellets (84.052 ng/ml) and the T_max_ of ER pellets (5.116 h) was higher than the IR pellets (3.029 h). No significant food effects were impacted the key PK parameters of ER pellets. Thus, the use of ammonio methacrylate copolymer, type A (RL100) coated pellets of Meclizine HCl is proposed to achieve required/substantial therapeutic effect for an extended period of time with comparable pharmacokinetics to immediate-release pellets in terms of AUC_0–t_ and AUC_0–∞_.

## Supplementary information

Supplementary Legends.

Supplementary Figure S1.

Supplementary Figure S2.

Supplementary Figure S3.

Supplementary Figure S4.

Supplementary Figure S5.

Supplementary Table S1.

Supplementary Table S2.

Supplementary Table S3.

Supplementary Table S4.

Supplementary Table S5.

Supplementary Table S6.
