# Supplementary material for: QbD based Eudragit coated Meclizine HCl immediate and extended release multiparticulates: formulation, characterization and pharmacokinetic evaluation using HPLC-Fluorescence detection method
Source: Sci Rep. 2020 Sep 10;10:14765. doi: 10.1038/s41598-020-71751-y (PMC7484796; doi:10.1038/s41598-020-71751-y)
Supplement: Supplementary file 1 — Supplementary Legends. [file 41598_2020_71751_MOESM1_ESM.docx]

**Figure S1** Structure of diphenylmethane chromophore.

**Figure S2** FTIR spectra showing (a) pure Meclizine (b) IR drug core pellets (c) Eudragit RL100 coated pellets (d) Eudragit RS100 coated pellets.

**Figure S3** EDS spectra showing elemental composition of (a) IR drug core pellets, (b) Eudragit RL100 coated pellets and (c) Eudragit RS100 coated pellets.

**Figure S4** Pre-treatment of plasma samples by (a & b) liquid extraction (c & d) liquid extraction in two steps (e & f) double extraction (g & h) protein precipitation with 5 min heating (i & j) protein precipitation with 10 min heating (k & l) protein precipitation with acetonitrile in different ratios to plasma (0.9:1).

**Figure S5** Extraction of Meclizine from plasma with other fluorescent drugs (a) Flunarizine (b) Cinnarizine (c) Pyridoxine (d) Levofloxacin (e) Pefloxacin (f) Ofloxacin.

**Table S1.** Image analysis of Meclizine HCl ER pellets coated with Eudragit RL100 and RS100.

**Table S2.** Kinetic parameters for dissolution data of Meclizine HCl ER Eudragit RL100 coated pellet formulations according to various kinetic models.

Table S3. Kinetic parameters for dissolution data of Meclizine HCl ER Eudragit RS100 coated pellet formulations according to various kinetic models.

**Table S4.** Intraday and interday accuracy and precision of Meclizine in plasma.

**Table S5.** Chromatographic variables and their responses investigated during robustness evaluation of the developed HPLC-Fluorescence method

Table S6. Details of volunteers participated in Part 1(single dose IR vs. ER) and Part 2 (single dose ER pellets food effect) studies.
