# Supplementary figures and images for: QbD based Eudragit coated Meclizine HCl immediate and extended release multiparticulates: formulation, characterization and pharmacokinetic evaluation using HPLC-Fluorescence detection method
Source: Sci Rep. 2020 Sep 10;10:14765. doi: 10.1038/s41598-020-71751-y (PMC7484796; doi:10.1038/s41598-020-71751-y)

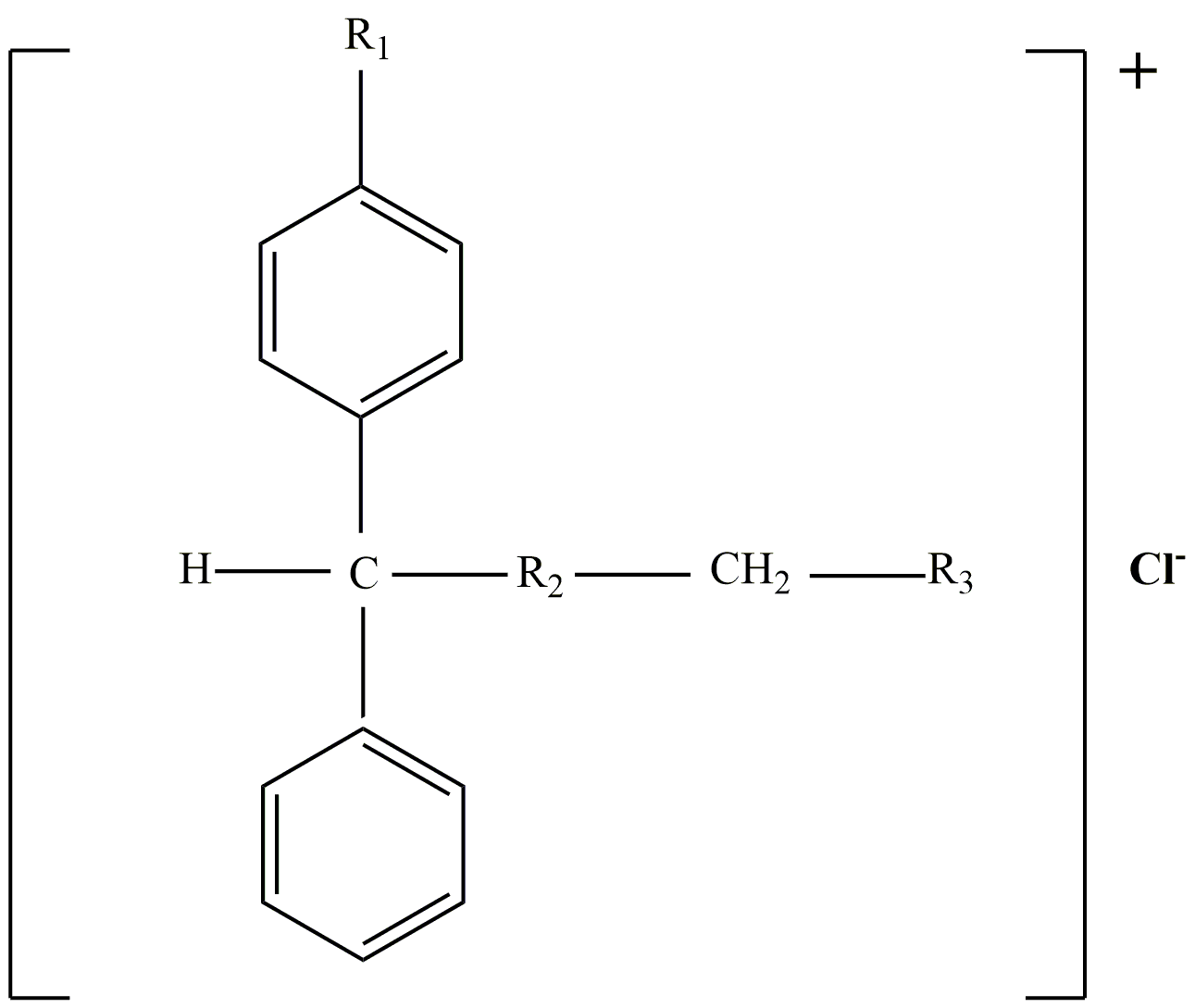

Supplement: Supplementary file 2 — Supplementary Figure S1. [file 41598_2020_71751_MOESM2_ESM.png]

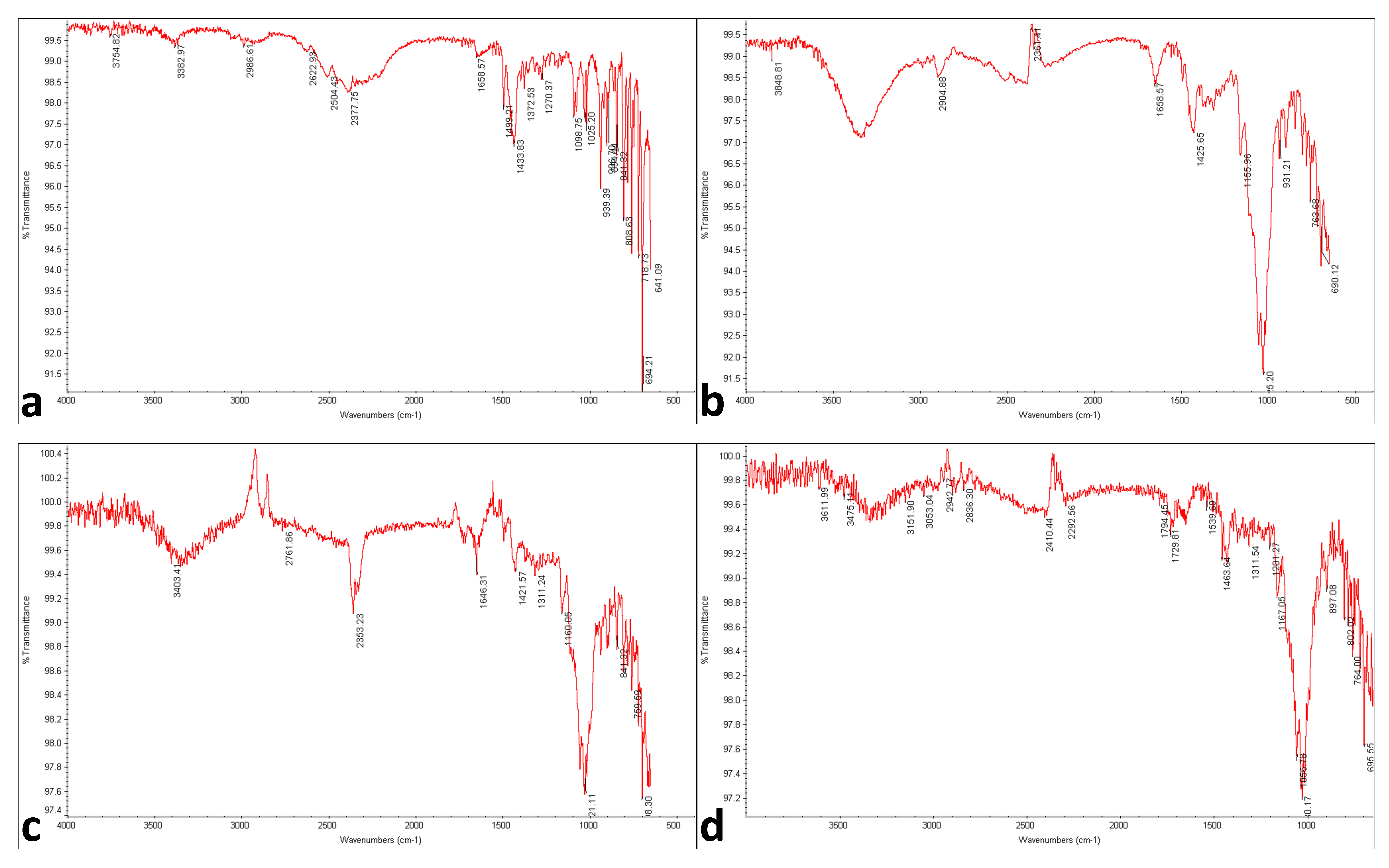

Supplement: Supplementary file 3 — Supplementary Figure S2. [file 41598_2020_71751_MOESM3_ESM.png]

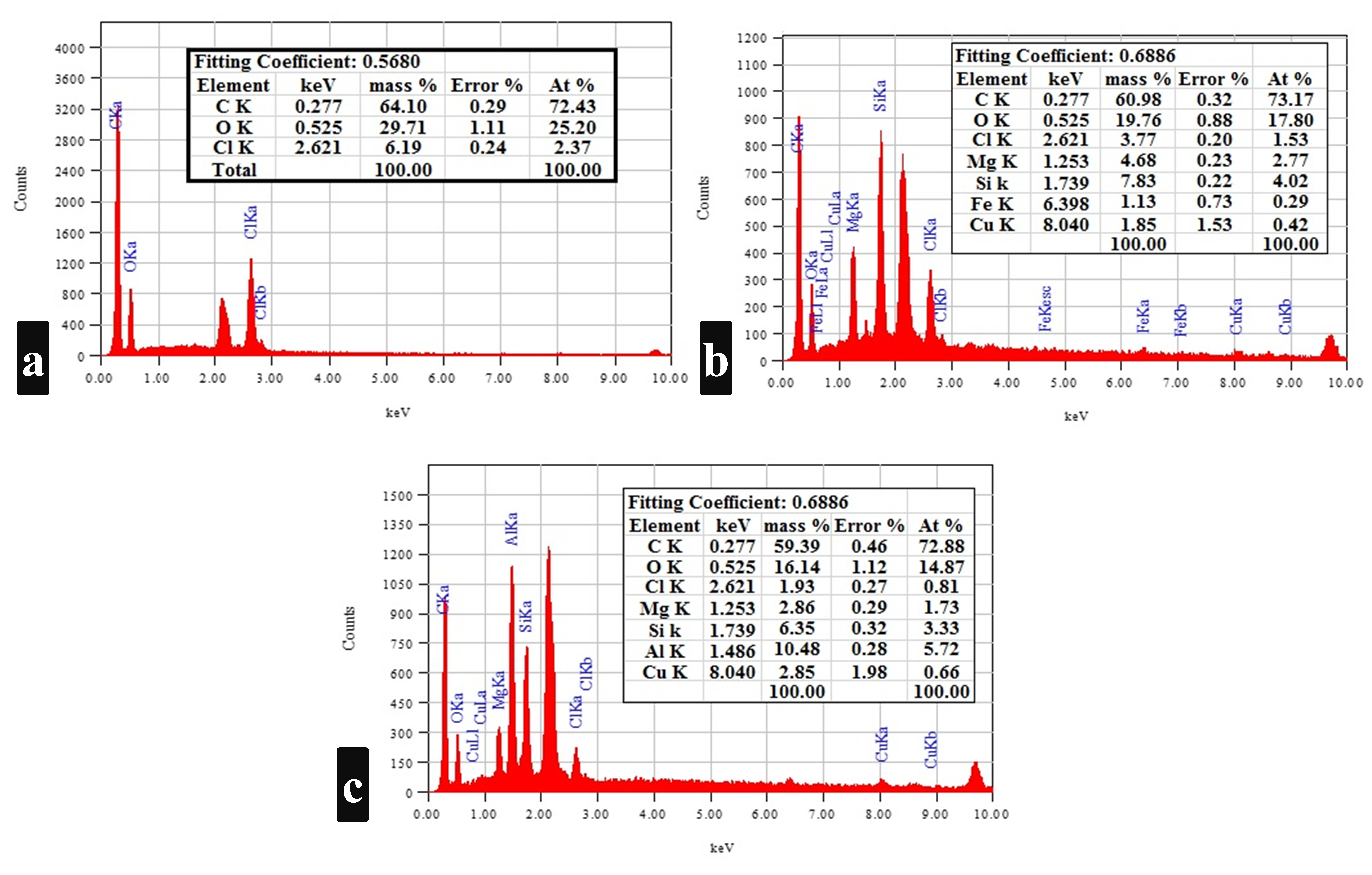

Supplement: Supplementary file 4 — Supplementary Figure S3. [file 41598_2020_71751_MOESM4_ESM.png]

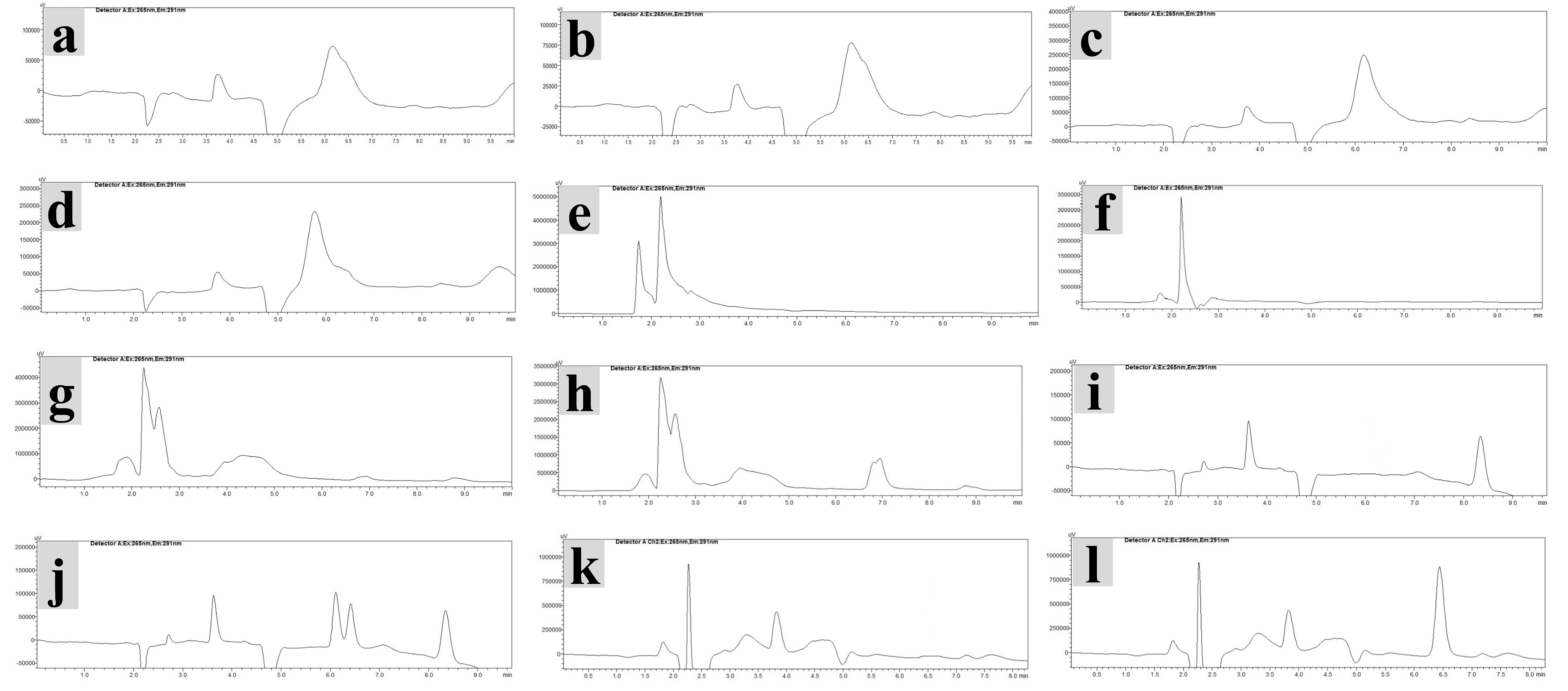

Supplement: Supplementary file 5 — Supplementary Figure S4. [file 41598_2020_71751_MOESM5_ESM.png]

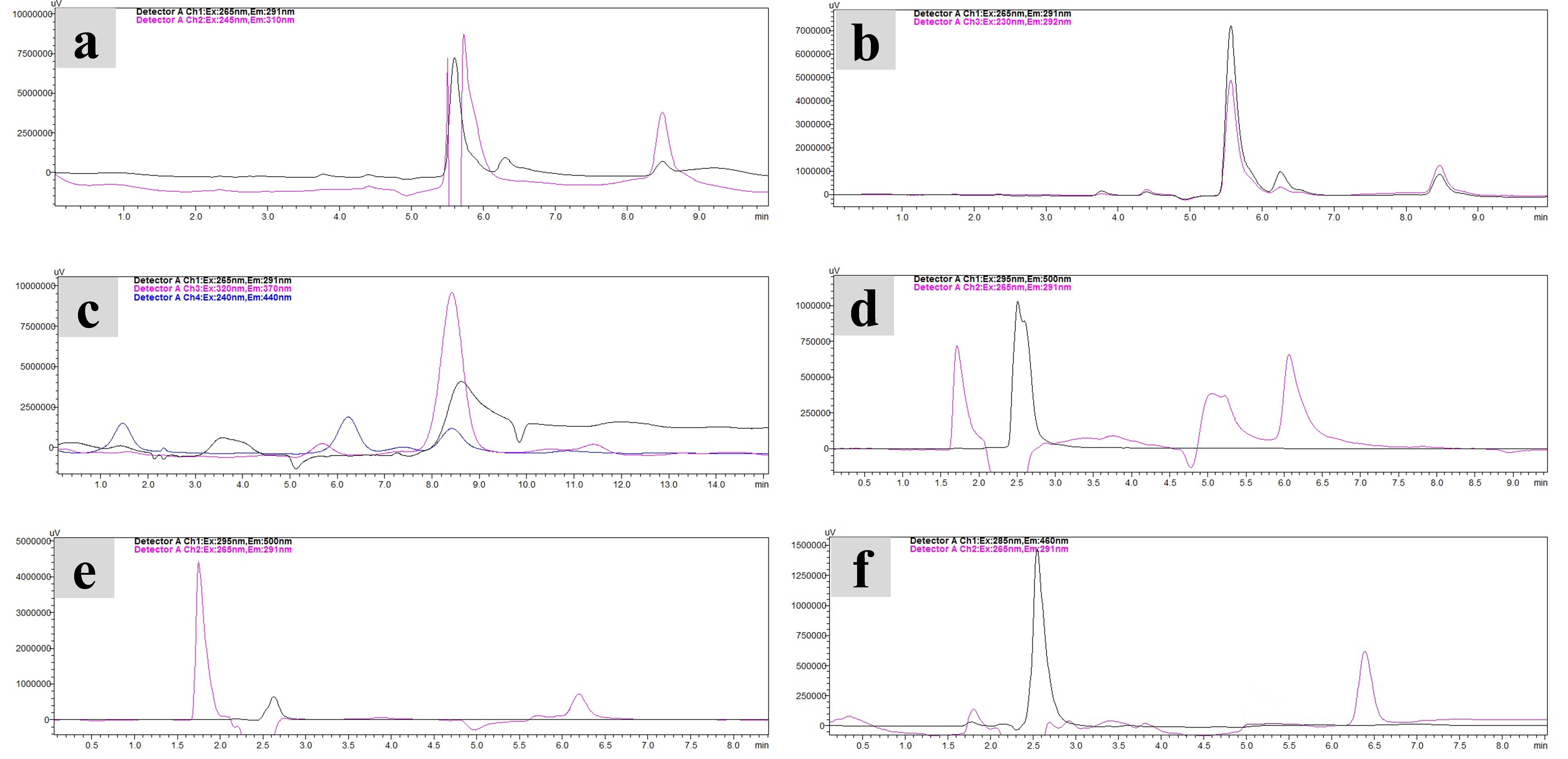

Supplement: Supplementary file 6 — Supplementary Figure S5. [file 41598_2020_71751_MOESM6_ESM.png]
