# Supplementary material for: QbD based Eudragit coated Meclizine HCl immediate and extended release multiparticulates: formulation, characterization and pharmacokinetic evaluation using HPLC-Fluorescence detection method
Source: Sci Rep. 2020 Sep 10;10:14765. doi: 10.1038/s41598-020-71751-y (PMC7484796; doi:10.1038/s41598-020-71751-y)
Supplement: Supplementary file 7 — Supplementary Table S1. [file 41598_2020_71751_MOESM7_ESM.docx]

| **Codes** | **Area** | **Perimeter** | | **Feret Diameter** | **Circularity** | **PS** | **Dce** | **ER** | **AR** | **eR** |
| --- | --- | --- | --- | --- | --- | --- | --- | --- | --- | --- |
| **Eudragit^®^ RL100 coated Pellets** | | | | | | | | | | |
| FC11 | 23865 | 588.907 | 180.945 | | 0.897 | 0.932 | 174.360 | 1.000 | 1.018 | 0.975 |
| FC12 | 25182 | 607.198 | 189.021 | | 0.912 | 0.927 | 179.106 | 1.000 | 1.020 | 0.914 |
| FC13 | 25845 | 610.528 | 189.381 | | 0.881 | 0.890 | 181.449 | 0.987 | 1.020 | 0.891 |
| FC14 | 25906 | 614.006 | 190.205 | | 0.858 | 0.851 | 181.663 | 0.979 | 1.020 | 0.866 |
| FC15 | 26156 | 615.831 | 191.212 | | 0.890 | 0.892 | 182.537 | 0.984 | 1.020 | 0.878 |
| FC16 | 27132 | 616.966 | 194.487 | | 0.896 | 0.891 | 185.911 | 0.981 | 1.085 | 0.807 |
| FC17 | 27132 | 616.966 | 194.487 | | 0.896 | 0.891 | 185.911 | 0.981 | 1.085 | 0.807 |
| FC18 | 27132 | 616.966 | 194.487 | | 0.896 | 0.891 | 185.911 | 0.981 | 1.085 | 0.807 |
| FC19 | 27132 | 616.966 | 194.487 | | 0.896 | 0.891 | 185.911 | 0.981 | 1.085 | 0.807 |
| FC20 | 27132 | 616.966 | 194.487 | | 0.896 | 0.891 | 185.911 | 0.981 | 1.085 | 0.807 |
| FC21 | 27132 | 616.966 | 194.487 | | 0.896 | 0.891 | 185.911 | 0.981 | 1.085 | 0.807 |
| FC22 | 27134 | 617.606 | 194.538 | | 0.904 | 0.908 | 185.918 | 0.971 | 1.085 | 0.774 |
| FC23 | 27266 | 617.313 | 195.064 | | 0.898 | 0.901 | 186.370 | 0.973 | 1.085 | 0.782 |
| FC24 | 27344 | 618.993 | 195.201 | | 0.945 | 0.929 | 186.636 | 0.976 | 1.085 | 0.795 |
| FC25 | 27419 | 619.158 | 195.747 | | 0.965 | 0.911 | 186.892 | 0.968 | 1.085 | 0.763 |
| FC26 | 27568 | 619.236 | 195.788 | | 0.909 | 0.896 | 187.399 | 0.965 | 1.072 | 0.743 |
| FC27 | 27634 | 620.479 | 196.163 | | 0.932 | 0.876 | 187.623 | 0.965 | 1.072 | 0.751 |
| FC28 | 27908 | 620.792 | 196.209 | | 0.944 | 0.921 | 188.551 | 0.958 | 1.072 | 0.714 |
| FC29 | 27913 | 621.361 | 197.331 | | 0.955 | 0.918 | 188.568 | 0.963 | 1.072 | 0.731 |
| FC30 | 28065 | 621.555 | 198.789 | | 0.920 | 0.902 | 189.081 | 0.962 | 1.085 | 0.723 |
| **Eudragit^®^ RS100 Coated Pellets** | | | | | | | | | | |
| FC31 | 26003 | 588.190 | 187.547 | | 0.944 | 0.892 | 182.002 | 0.986 | 1.066 | 0.873 |
| FC32 | 26233 | 593.827 | 188.469 | | 0.912 | 0.848 | 182.805 | 0.982 | 1.048 | 0.855 |
| FC33 | 26414 | 594.053 | 189.191 | | 0.934 | 0.842 | 183.435 | 0.980 | 1.048 | 0.851 |
| FC34 | 26659 | 594.253 | 190.013 | | 0.929 | 0.827 | 184.284 | 0.972 | 1.048 | 0.814 |
| FC35 | 26931 | 595.266 | 191.002 | | 0.909 | 0.837 | 185.222 | 0.978 | 1.048 | 0.836 |
| FC36 | 27107 | 596.053 | 191.191 | | 0.934 | 0.839 | 185.435 | 0.979 | 1.042 | 0.838 |
| FC37 | 27107 | 596.053 | 191.191 | | 0.934 | 0.839 | 185.435 | 0.979 | 1.042 | 0.838 |
| FC38 | 27107 | 596.053 | 191.191 | | 0.934 | 0.839 | 185.435 | 0.979 | 1.042 | 0.838 |
| FC39 | 27107 | 596.053 | 191.191 | | 0.934 | 0.839 | 185.435 | 0.979 | 1.042 | 0.838 |
| FC40 | 27107 | 596.053 | 191.191 | | 0.934 | 0.839 | 185.435 | 0.979 | 1.042 | 0.838 |
| FC41 | 27107 | 596.053 | 191.191 | | 0.934 | 0.839 | 185.435 | 0.979 | 1.042 | 0.838 |
| FC42 | 27109 | 600.593 | 193.259 | | 0.966 | 0.829 | 185.833 | 0.972 | 1.042 | 0.816 |
| FC43 | 27487 | 609.675 | 194.538 | | 0.929 | 0.832 | 187.124 | 0.975 | 1.042 | 0.830 |
| FC44 | 27634 | 610.528 | 196.840 | | 0.932 | 0.835 | 187.623 | 0.977 | 1.042 | 0.833 |
| FC45 | 27802 | 615.249 | 197.085 | | 0.915 | 0.821 | 188.193 | 0.969 | 1.042 | 0.812 |
| FC46 | 28095 | 617.717 | 187.547 | | 0.909 | 0.817 | 189.182 | 0.964 | 1.061 | 0.786 |
| FC47 | 28352 | 618.082 | 188.469 | | 0.934 | 0.820 | 190.045 | 0.965 | 1.061 | 0.799 |
| FC48 | 28451 | 620.057 | 189.191 | | 0.919 | 0.815 | 190.377 | 0.962 | 1.061 | 0.780 |
| FC49 | 28652 | 623.205 | 190.013 | | 0.936 | 0.819 | 191.048 | 0.965 | 1.061 | 0.797 |
| FC50 | 28948 | 623.716 | 191.002 | | 0.885 | 0.813 | 192.032 | 0.960 | 1.061 | 0.758 |

**Table S1:** Image analysis of Meclizine HCl ER pellets coated with Eudragit^®^ RL100 and RS100.

**Note:** PS = Projection sphericity, Dce = Circle equivalent diameter, ER = Elongation ratio, AR = Aspect ratio, eR=Two dimensional shape factor
