# Supplementary material for: QbD based Eudragit coated Meclizine HCl immediate and extended release multiparticulates: formulation, characterization and pharmacokinetic evaluation using HPLC-Fluorescence detection method
Source: Sci Rep. 2020 Sep 10;10:14765. doi: 10.1038/s41598-020-71751-y (PMC7484796; doi:10.1038/s41598-020-71751-y)
Supplement: Supplementary file 8 — Supplementary Table S2. [file 41598_2020_71751_MOESM8_ESM.docx]

| **Codes** | **Zero-order** | | **First-order** | | **Higuchi’s** | | **Hixson-Crowell** | | **Baker-Lonsdale** | | **Jander’s** | | **Korsmeyer-Peppas** | | | **MDT** | **DE_6_** |
| --- | --- | --- | --- | --- | --- | --- | --- | --- | --- | --- | --- | --- | --- | --- | --- | --- | --- |
|  | **R^2^** | **K_0_**  **(h^-1^)** | **R^2^** | **K_1_**  **(h^-1^)** | **R^2^** | **K_H_**  **(h^-1/2^)** | **R^2^** | **K_HC_ (h^-1^)** | **R^2^** | **K_BL_ (h^-1^)** | **R^2^** | **K_J_**  **(h^-1/2^)** | **R^2^** | **n** | **K_KP_ (h^-n^)** | **h** | **%** |
| **FC11** | 0.987 | 11.462 | 0.836 | 0.266 | 0.988 | 41.272 | 0.957 | 0.957 | 0.906 | 0.033 | 0.878 | 0.222 | 0.991 | 0.860 | 0.165 | 3.270 | N/A |
| **FC12** | 0.978 | 6.903 | 0.867 | 0.122 | 0.977 | 29.75 | 0.945 | 0.197 | 0.856 | 0.012 | 0.880 | 0.112 | 0.992 | 0.749 | 0.146 | 5.366 | 29.920 |
| **FC13** | 0.986 | 7.005 | 0.896 | 0.116 | 0.977 | 30.087 | 0.958 | 0.201 | 0.876 | 0.011 | 0.888 | 0.108 | 0.991 | 0.735 | 0.150 | 5.302 | 29.619 |
| **FC14** | 0.987 | 6.909 | 0.925 | 0.110 | 0.978 | 29.668 | 0.972 | 0.197 | 0.904 | 0.010 | 0.905 | 0.102 | 0.989 | 0.708 | 0.158 | 5.146 | 29.456 |
| **FC15** | 0.987 | 6.907 | 0.927 | 0.114 | 0.980 | 29.677 | 0.971 | 0.190 | 0.893 | 0.010 | 0.906 | 0.106 | 0.994 | 0.710 | 0.155 | 5.283 | 29.291 |
| **FC16** | 0.974 | 6.973 | 0.978 | 0.106 | 0.961 | 29.866 | 0.985 | 0.176 | 0.947 | 0.008 | 0.932 | 0.080 | 0.944 | 0.704 | 0.142 | 5.083 | 31.904 |
| **FC17** | 0.974 | 6.973 | 0.978 | 0.106 | 0.961 | 29.866 | 0.985 | 0.176 | 0.947 | 0.008 | 0.932 | 0.080 | 0.944 | 0.704 | 0.142 | 5.083 | 31.904 |
| **FC18** | 0.974 | 6.973 | 0.978 | 0.106 | 0.961 | 29.866 | 0.985 | 0.176 | 0.947 | 0.008 | 0.932 | 0.080 | 0.944 | 0.704 | 0.142 | 5.083 | 31.904 |
| **FC19** | 0.974 | 6.973 | 0.978 | 0.106 | 0.961 | 29.866 | 0.985 | 0.176 | 0.947 | 0.008 | 0.932 | 0.080 | 0.944 | 0.704 | 0.142 | 5.083 | 31.904 |
| **FC20** | 0.974 | 6.973 | 0.978 | 0.106 | 0.961 | 29.866 | 0.985 | 0.176 | 0.947 | 0.008 | 0.932 | 0.080 | 0.944 | 0.704 | 0.142 | 5.083 | 31.904 |
| **FC21** | 0.974 | 6.973 | 0.978 | 0.106 | 0.961 | 29.866 | 0.985 | 0.176 | 0.947 | 0.008 | 0.932 | 0.080 | 0.944 | 0.704 | 0.142 | 5.083 | 31.904 |
| **FC22** | 0.984 | 6.724 | 0.983 | 0.110 | 0.969 | 28.782 | 0.992 | 0.167 | 0.948 | 0.009 | 0.937 | 0.083 | 0.957 | 0.696 | 0.143 | 5.128 | 30.739 |
| **FC23** | 0.989 | 6.442 | 0.982 | 0.100 | 0.973 | 27.561 | 0.993 | 0.157 | 0.943 | 0.008 | 0.938 | 0.094 | 0.969 | 0.707 | 0.138 | 5.206 | 31.068 |
| **FC24** | 0.989 | 6.442 | 0.982 | 0.100 | 0.973 | 27.561 | 0.993 | 0.157 | 0.943 | 0.008 | 0.938 | 0.094 | 0.969 | 0.755 | 0.122 | 5.206 | 31.068 |
| **FC25** | 0.983 | 6.589 | 0.987 | 0.1014 | 0.974 | 28.292 | 0.993 | 0.165 | 0.958 | 0.008 | 0.945 | 0.096 | 0.971 | 0.725 | 0.132 | 4.888 | 31.475 |
| **FC26** | 0.972 | 5.529 | 0.907 | 0.0441 | 0.894 | 22.873 | 0.934 | 0.128 | 0.831 | 0.003 | 0.831 | 0.043 | 0.918 | 0.769 | 0.111 | 5.974 | 20.629 |
| **FC27** | 0.972 | 5.529 | 0.907 | 0.0441 | 0.894 | 22.873 | 0.934 | 0.128 | 0.831 | 0.003 | 0.831 | 0.043 | 0.918 | 0.769 | 0.142 | 5.974 | 20.629 |
| **FC28** | 0.959 | 5.323 | 0.901 | 0.0379 | 0.867 | 21.833 | 0.925 | 0.121 | 0.825 | 0.002 | 0.814 | 0.035 | 0.837 | 0.758 | 0.112 | 6.141 | 19.496 |
| **FC29** | 0.974 | 5.319 | 0.925 | 0.0369 | 0.898 | 22.03 | 0.947 | 0.120 | 0.854 | 0.002 | 0.849 | 0.036 | 0.866 | 0.763 | 0.109 | 5.923 | 26.597 |
| **FC30** | 0.989 | 4.767 | 0.956 | 0.0198 | 0.928 | 19.916 | 0.970 | 0.103 | 0.885 | 0.002 | 0.887 | 0.032 | 0.940 | 0.743 | 0.103 | 5.680 | 24.080 |

**Table S2:** Kinetic parameters for dissolution data of Meclizine HCl ER Eudragit^®^ RL100 coated pellet formulations according to various kinetic models.

Note: "R^2^ " is the regression coefficient; "K" is the release rate constant for respective models; "n" is the diffusion exponent;"MDT" is the mean dissolution time, "DE" is the dissolution efficiency at 6h. N/A indicates that more than 90% drug released within 1-2h.
