# Supplementary material for: QbD based Eudragit coated Meclizine HCl immediate and extended release multiparticulates: formulation, characterization and pharmacokinetic evaluation using HPLC-Fluorescence detection method
Source: Sci Rep. 2020 Sep 10;10:14765. doi: 10.1038/s41598-020-71751-y (PMC7484796; doi:10.1038/s41598-020-71751-y)
Supplement: Supplementary file 9 — Supplementary Table S3. [file 41598_2020_71751_MOESM9_ESM.docx]

**Table S3:** Kinetic parameters for dissolution data of Meclizine HCl ER Eudragit^®^ RS100 coated pellet formulations according to various kinetic models

| **Codes** | **Zero-order** | | **First-order** | | **Higuchi’s** | | **Hixson-Crowell** | | **Baker-Lonsdale** | | **Jander’s** | | **Korsmeyer-Peppas** | | | **MDT** | **DE6** |
| --- | --- | --- | --- | --- | --- | --- | --- | --- | --- | --- | --- | --- | --- | --- | --- | --- | --- |
|  | **R^2^** | **K_0_**  **(h^-1^)** | **R^2^** | **K_1_**  **(h^-1^)** | **R^2^** | **K_H_**  **(h^-1/2^)** | **R^2^** | **K_HC_ (h^-1^)** | **R^2^** | **K_BL_ (h^-1^)** | **R^2^** | **K_J_**  **(h^-1/2^)** | **R^2^** | **n** | **K_KP_ (h^-n^)** | **h** | **%** |
| **FC31** | 0.983 | 6.006 | 0.951 | 0.110 | 0.989 | 25.983 | 0.979 | 0.154 | 0.910 | 0.010 | 0.934 | 0.104 | 0.998 | 0.670 | 0.159 | 5.138 | 30.008 |
| **FC32** | 0.979 | 5.773 | 0.956 | 0.093 | 0.978 | 24.895 | 0.974 | 0.134 | 0.891 | 0.007 | 0.932 | 0.091 | 0.994 | 0.811 | 0.101 | 5.411 | 31.461 |
| **FC33** | 0.986 | 5.849 | 0.966 | 0.086 | 0.980 | 25.157 | 0.982 | 0.135 | 0.904 | 0.006 | 0.936 | 0.084 | 0.993 | 0.712 | 0.127 | 5.396 | 30.238 |
| **FC34** | 0.988 | 5.713 | 0.975 | 0.087 | 0.985 | 24.606 | 0.988 | 0.130 | 0.918 | 0.006 | 0.946 | 0.084 | 0.996 | 0.762 | 0.111 | 5.369 | 30.435 |
| **FC35** | 0.987 | 5.727 | 0.989 | 0.088 | 0.989 | 24.740 | 0.995 | 0.129 | 0.945 | 0.006 | 0.961 | 0.085 | 0.996 | 0.791 | 0.101 | 5.239 | 31.399 |
| **FC36** | 0.987 | 5.622 | 0.987 | 0.074 | 0.979 | 24.158 | 0.992 | 0.123 | 0.939 | 0.006 | 0.953 | 0.069 | 0.985 | 0.768 | 0.104 | 5.460 | 30.614 |
| **FC37** | 0.987 | 5.622 | 0.987 | 0.074 | 0.979 | 24.158 | 0.992 | 0.123 | 0.939 | 0.006 | 0.953 | 0.069 | 0.985 | 0.768 | 0.104 | 5.460 | 30.614 |
| **FC38** | 0.987 | 5.622 | 0.987 | 0.074 | 0.979 | 24.158 | 0.992 | 0.123 | 0.939 | 0.006 | 0.953 | 0.069 | 0.985 | 0.768 | 0.104 | 5.460 | 30.614 |
| **FC39** | 0.987 | 5.622 | 0.987 | 0.074 | 0.979 | 24.158 | 0.992 | 0.123 | 0.939 | 0.006 | 0.953 | 0.069 | 0.985 | 0.768 | 0.104 | 5.460 | 30.614 |
| **FC40** | 0.987 | 5.622 | 0.987 | 0.074 | 0.979 | 24.158 | 0.992 | 0.123 | 0.939 | 0.006 | 0.953 | 0.069 | 0.985 | 0.768 | 0.104 | 5.460 | 30.614 |
| **FC41** | 0.987 | 5.622 | 0.987 | 0.074 | 0.979 | 24.158 | 0.992 | 0.123 | 0.939 | 0.006 | 0.953 | 0.069 | 0.985 | 0.768 | 0.104 | 5.460 | 30.614 |
| **FC42** | 0.985 | 5.614 | 0.978 | 0.068 | 0.976 | 24.114 | 0.987 | 0.125 | 0.925 | 0.006 | 0.945 | 0.072 | 0.992 | 0.809 | 0.095 | 5.458 | 30.773 |
| **FC43** | 0.993 | 5.572 | 0.991 | 0.078 | 0.980 | 23.889 | 0.996 | 0.123 | 0.944 | 0.005 | 0.952 | 0.076 | 0.996 | 0.802 | 0.094 | 5.303 | 29.403 |
| **FC44** | 0.996 | 5.729 | 0.985 | 0.073 | 0.975 | 24.445 | 0.994 | 0.127 | 0.929 | 0.004 | 0.940 | 0.073 | 0.993 | 0.801 | 0.097 | 5.498 | 29.947 |
| **FC45** | 0.995 | 5.570 | 0.988 | 0.072 | 0.973 | 23.769 | 0.995 | 0.122 | 0.939 | 0.004 | 0.944 | 0.070 | 0.993 | 0.759 | 0.083 | 5.396 | 27.542 |
| **FC46** | 0.976 | 4.179 | 0.944 | 0.039 | 0.923 | 17.529 | 0.958 | 0.086 | 0.858 | 0.002 | 0.883 | 0.038 | 0.959 | 0.718 | 0.099 | 5.800 | 22.832 |
| **FC47** | 0.986 | 4.277 | 0.960 | 0.038 | 0.937 | 17.992 | 0.971 | 0.088 | 0.884 | 0.002 | 0.901 | 0.038 | 0.971 | 0.706 | 0.101 | 5.622 | 24.786 |
| **FC48** | 0.989 | 4.206 | 0.971 | 0.037 | 0.950 | 17.787 | 0.980 | 0.085 | 0.899 | 0.002 | 0.919 | 0.038 | 0.976 | 0.719 | 0.096 | 5.708 | 24.277 |
| **FC49** | 0.972 | 3.866 | 0.944 | 0.039 | 0.899 | 16.036 | 0.956 | 0.079 | 0.875 | 0.002 | 0.867 | 0.036 | 0.906 | 0.718 | 0.094 | 5.643 | 22.730 |
| **FC50** | 0.987 | 3.586 | 0.978 | 0.029 | 0.964 | 15.287 | 0.983 | 0.070 | 0.912 | 0.002 | 0.939 | 0.036 | 0.984 | 0.739 | 0.080 | 5.402 | 26.379 |
