# Supplementary material for: QbD based Eudragit coated Meclizine HCl immediate and extended release multiparticulates: formulation, characterization and pharmacokinetic evaluation using HPLC-Fluorescence detection method
Source: Sci Rep. 2020 Sep 10;10:14765. doi: 10.1038/s41598-020-71751-y (PMC7484796; doi:10.1038/s41598-020-71751-y)
Supplement: Supplementary file 10 — Supplementary Table S4. [file 41598_2020_71751_MOESM10_ESM.docx]

**Table S4:** Intraday and interday accuracy and precision of Meclizine in plasma

| **Conc. (ng/ml)** | **Intraday (n=5)** | | | **Interday (n=5)** | | |
| --- | --- | --- | --- | --- | --- | --- |
|  | **Mean ± SD** | **Accuracy** | **Precision (CV)** | **Mean ± SD** | **Accuracy** | **Precision**  **(CV)** |
| 10 | 10.032 ± 0.026 | 100.077 | 0.261 | 10.116 ± 0.072 | 100.984 | 0.713 |
| 30 | 30.216 ± 0.011 | 101.689 | 0.037 | 29.9576 ± 0.272 | 99.867 | 0.908 |
| 100 | 100.184 ± 0.177 | 101.053 | 0.177 | 100.157 ± 0.187 | 100.092 | 0.187 |
| 180 | 179.976 ± 0.171 | 99.941 | 0.094 | 180.068 ± 0.449 | 100.048 | 0.249 |
| 200 | 199.797 ± 0.494 | 99.952 | 0.247 | 200.276 ± 0.266 | 100.751 | 0.133 |
