# Supplementary material for: QbD based Eudragit coated Meclizine HCl immediate and extended release multiparticulates: formulation, characterization and pharmacokinetic evaluation using HPLC-Fluorescence detection method
Source: Sci Rep. 2020 Sep 10;10:14765. doi: 10.1038/s41598-020-71751-y (PMC7484796; doi:10.1038/s41598-020-71751-y)
Supplement: Supplementary file 11 — Supplementary Table S5. [file 41598_2020_71751_MOESM11_ESM.docx]

**Table S5:** Chromatographic variables and their responses investigated during robustness evaluation of the developed HPLC-Fluorescence method

| **Variables** | **Responses** | | | | | |
| --- | --- | --- | --- | --- | --- | --- |
|  | **Meclizine** | | | **Ofloxacin** | | |
|  | **Level**  **(-1, 0, 1)** | **Recovery Mean**  **(%)** | **Retention time**  **(min)** | **Level**  **(-1, 0, 1)** | **Recovery Mean**  **(%)** | **Retention time**  **(min)** |
| **Flow Rate (ml/min)** | 0.9 | 100.149 ± 0.449 | 6.4 ± 0.109 | 0.9 | 101.241 ± 0.921 | 2.6 ± 0.119 |
|  | 1.0 | 100.882 ± 0.908 | 6.2 ± 0.211 | 1.0 | 100.723 ± 1.256 | 2.5 ± 0.187 |
|  | 1.1 | 98.976 ± 0.541 | 6.1 ± 0.134 | 1.1 | 99.986 ± 1.834 | 2.4 ± 0.125 |
| **pH** | 2.5 | 101.586 ± 0.972 | 6.3 ± 0.110 | 2.5 | 99.775 ± 0.792 | 2.6 ± 0.104 |
|  | 3.0 | 99.954 ± 0.631 | 6.3 ± 0.110 | 3.0 | 100.239 ± 1.471 | 2.5 ± 0.203 |
|  | 3.5 | 98.682 ± 0.729 | 6.1 ± 0.120 | 3.5 | 100.343 ± 0.821 | 2.6 ± 0.115 |
| **Buffer: ACN** | 295:705 | 101.023 ± 1.885 | 6.4 ± 0.108 | 295:705 | 99.911 ± 1.524 | 2.6 ± 0.109 |
|  | 300:700 | 100.126 ± 0.971 | 6.3 ± 0.121 | 300:700 | 100.019 ± 1.842 | 2.5 ± 0.197 |
|  | 305:695 | 99.319 ± 1.032 | 6.1 ± 0.143 | 305:695 | 100.519 ± 0.928 | 2.4 ± 0.146 |
| **Excitation Wavelength**  **nm** | 262 | 99.895 ± 1.325 | 6.3 ± 0.113 | 282 | 98.965 ± 1.193 | 2.6 ± 0.119 |
|  | 265 | 100.291 ± 0.872 | 6.2 ± 0.208 | 285 | 100.741 ± 1.501 | 2.5 ± 0.207 |
|  | 268 | 101.734 ± 1.056 | 6.1 ± 0.148 | 288 | 101.092 ± 0.928 | 2.5 ± 0.213 |
| **Emission Wavelength**  **nm** | 288 | 99.861 ± 1.407 | 6.2 ± 0.199 | 457 | 99.764 ± 1.128 | 2.5 ± 0.224 |
|  | 291 | 100.076 ± 0.951 | 6.2 ± 0.201 | 460 | 100.533 ± 1.245 | 2.5 ± 0.192 |
|  | 294 | 100.251± 1.108 | 6.3 ± 0.130 | 463 | 101.818 ± 1.034 | 2.6 ± 0.118 |
