# Supplementary material for: QbD based Eudragit coated Meclizine HCl immediate and extended release multiparticulates: formulation, characterization and pharmacokinetic evaluation using HPLC-Fluorescence detection method
Source: Sci Rep. 2020 Sep 10;10:14765. doi: 10.1038/s41598-020-71751-y (PMC7484796; doi:10.1038/s41598-020-71751-y)
Supplement: Supplementary file 12 — Supplementary Table S6. [file 41598_2020_71751_MOESM12_ESM.docx]

Table S6: Details of volunteers participated in Part 1 (single dose IR vs. ER) and Part 2 (single dose ER pellets food effect) studies

| **Part 1 (single dose IR vs. ER)** | | | | | **Part 2 (single dose ER pellets food effect)** | | | | |
| --- | --- | --- | --- | --- | --- | --- | --- | --- | --- |
| **Volunteer code** | **Sequence** | **Age** | **Weight** | **Height** | **Volunteer code** | **Sequence** | **Age** | **Weight** | **Height** |
|  |  | **Year** | **Kg** | **ft. in** |  |  | **Year** | **Kg** | **ft. in** |
| V1 | ERIR | 22 | 66 | 5’5” | V1 | FedFast | 24 | 69 | 5’11” |
| V2 | IRER | 22 | 68 | 5’9” | V2 | FastFed | 24 | 68 | 5’9” |
| V3 | IRER | 23 | 78 | 5’8” | V3 | FastFed | 23 | 66 | 5’10” |
| V4 | ERIR | 23 | 80 | 5’8” | V4 | FedFast | 22 | 74 | 5’9” |
| V5 | IRER | 23 | 84 | 5’6” | V5 | FastFed | 22 | 61 | 5’11” |
| V6 | ERIR | 22 | 61 | 5’8” | V6 | FedFast | 22 | 62 | 5’9” |
| V7 | ERIR | 22 | 59 | 5’10” | V7 | FedFast | 23 | 85 | 5’7” |
| V8 | IRER | 23 | 73 | 5’8” | V8 | FastFed | 23 | 60 | 5.11” |
| V9 | ERIR | 23 | 60 | 5’10” | V9 | FedFast | 23 | 80 | 5’9” |
| V10 | IRER | 24 | 65 | 5’9” | V10 | FastFed | 23 | 79 | 5’9” |
| V11 | IRER | 24 | 67 | 5’8” | V11 | FastFed | 22 | 69 | 6’0” |
| V12 | ERIR | 22 | 68 | 5’10” | V12 | FedFast | 22 | 67 | 5’6” |
